# Tryptanthrin promotes keratinocyte and fibroblast responses *in vitro* after infection with *Trichophyton benhamiae* DSM6916

**DOI:** 10.1038/s41598-020-58773-2

**Published:** 2020-02-05

**Authors:** Jana Hesse-Macabata, Bianka Morgner, Peter Elsner, Uta-Christina Hipler, Cornelia Wiegand

**Affiliations:** Department of Dermatology, Jena University Hospital, Jena, Germany

**Keywords:** Fungi, Molecular biology

## Abstract

Exceedingly virulent pathogens and growing antimicrobial resistances require new therapeutic approaches. The zoophilic dermatophyte *Trichophyton benhamiae* causes highly inflammatory, cutaneous fungal infections. Recently, it could be shown that the plant-derived alkaloid tryptanthrin (TRP) exhibits strong anti-microbial activities against yeasts and dermatophytes. The aim of this study was to analyse the bioactivity of TRP under infectious conditions using an *in-vitro* dermatophytosis model employing fibroblasts and keratinocytes infected with *T. benhamiae* DSM6916. Analyses comprised determination of cell viability, effects on the innate immune response including expression and secretion of pro-inflammatory cytokines/chemokines as well as expression of various antimicrobial peptides (AMP), toll-like receptor (TLR) 2 and proliferation marker *MKI67*. *T. benhamiae* caused severe inflammation in the cutaneous cell models. TRP almost fully prevented *T. benhamiae*-derived damage of dermal fibroblasts and substantially reduced it in epidermal keratinocytes. A distinct down-regulation of the expression and secretion of pro-inflammatory cytokines was observed. Further, TRP promoted AMP expression, especially of *HBD2* and *HBD3*, in keratinocytes even without fungal presence. This study provides crucial evidence that TRP is not only a strong antifungal agent but also potentially modulates the innate immune response. This makes it interesting as a natural antimycotic drug for adjuvant treatment and prevention of fungal re-infection.

## Introduction

Dermatophytoses present a serious public health issue affecting 20–25% of the world’s population^1^. Especially zoophilic dermatophytes often cause acute, highly inflammatory, cutaneous infections in humans^2,3^.

In Germany the guinea pig-associated dermatophyte *Trichophyton benhamiae* pertain to one of the most common cause for fungal infections especially among children and adolescents^4,5^. Due to the growing mobility and migration, a pathogen shift with an emergent incidence of new fungi can be observed^6^, which in turn leads to an increasing application of broad-spectrum antimycotics. A new more virulent and potentially contagious *Trichophyton mentagrophytes* strain was recently isolated in India. Its origin seems to be unknown, but its occurrence is dramatically on the rise even replacing *T. rubrum* as the most common species causing superficial dermatophytosis in India^7,8^. With the rising prevalence of fungal infections, the emerging antimycotic resistance^9,10^ as well as the appearance of new and highly virulent pathogens novel therapeutic approaches are required. Hence, it is of great interest to find alternative, natural, antimycotically effective agents. In addition to exhibiting antimicrobial properties, it would be worthwhile if they also potentially influenced the immune response by e.g. promoting the defence and immune responses of cells against invading pathogens.

Plants possess secondary metabolites that are anti-microbial compounds accumulating in regions of pathogenic infection. These include phytochemicals such as flavonoids, alkaloids, terpenoids and others. Epigallocatechin gallate (EGCG), an active compound of green tea, was shown to enhance the anti-fungal effect of amphotericin B or fluconazole against antimycotic-susceptible and -resistant *C. albicans*^11^. The alkaloid indolo[2,1-b]quinazolin-6,12-dione (tryptanthrin, TRP) isolated from woad, *Isatis tinctoria L*. (Brassicaceae) is one promising phytochemical^12^ exhibiting anti-inflammatory^13–15^ and anti-microbial effects^16,17^ whereby its anti-dermatophytic activity appeared to be most efficient compared to that against various Candida species^18,19^ Consequently, the strong antimycotic property of TRP has drawn interest to investigate its applicability as a new natural source of antimycotic drugs for potential adjuvant anti-fungal therapy or preventive treatment. So far, only little is known about the cellular and molecular mechanism of action involved in these phytopharmacological properties of TRPs.

Hence, a previously described dermatophytosis model of *T. benhamiae* infection of dermal fibroblasts or epidermal keratinocytes^20^ was used to investigate the bioactivity and biocompatibility of TRP in terms of its anti-fungal activity and its possibly cell protective effect under co-culture-conditions. The potential impact of TRP on the innate immune response was an additional objective of this study, as both cutaneous cells participate in the infection-derived immune response^20^. Furthermore, the effect of TRP in the absence of fungal infection was examined.

## Results

### Anti-dermatophytic effect of TRP

**Figure 1:**
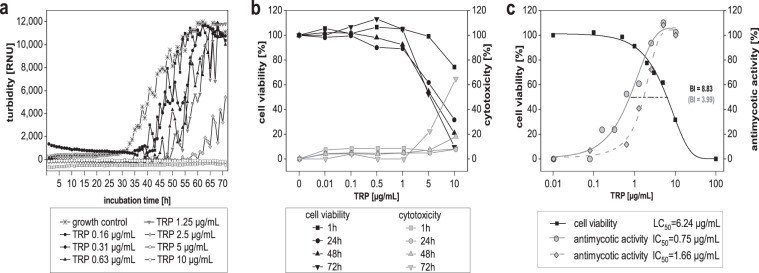
Antimicrobial activity and biocompatibility of tryptanthrin (TRP) and evaluation of its biocompatibility index (BI). The anti-microbial activity against *T. benhamiae* DSM6916 was analysed by means of the turbidity measurement using the microplate laser nephelometry (MLN). The fungal growth curves are represented in a concentration and time dependent manner (**a**). The biocompatibility includes the determination of the cell viability and the cytotoxicity using HaCaT keratinocytes treated with concentration series of TRP for 1 h, 24 h, 48 h and 72 h. Cell viability was analysed by measuring the cellular ATP level (black) and is presented as percentage to growth control (100%). Cytotoxicity [%] was evaluated by quantifying the LDH release (grey) is represented relative to lysis control (100%, data not shown) and growth control (0%) (**b**). The anti-microbial activity was further analysed by quantifying the fungal ATP content using the BacTiter^TM^-Glo assay (ATP_fungi_). In order to evaluate the BI of TRP the dose-response curves of the cell compatibility after 24 h (black square) and anti-microbial activity (MLN = grey circle, ATP_fungi_ = grey rhombus) was compared as the ratio of LC_50_ and IC_50_ (**c**).

The anti-dermatophytic activity of TRP was determined over a course of 72 h and revealed a fungicidal activity of TRP against *T. benhamiae* DSM6916, with a MIC of 4 µg/mL and an IC_50_-value of 0.75 µg/mL measured by microplate laser nephelometry (MLN). A slightly divergent IC_50_-value of 1.66 µg/mL was determined based on fungal ATP content (Fig. 1a). Effects of the solvent dimethyl sulfoxide (DMSO) were excluded in all experiments by testing DMSO alone. Here an IC_50_-value of 5.75% (see Supplementary Material Fig. S1) was determined, which is significantly higher than the corresponding DMSO concentrations of 0.075% and 0.166% in the TRP preparations, respectively.

In the dermatophytosis models of epidermal keratinocytes and dermal fibroblasts infected with *T. benhamiae* DSM6916, evaluation of the anti-dermatophytic effect was analysed based on the fluorescent intensity emitted by the calcofluor-stained chitin of the fungal cell wall. Tests indicated that already a low TRP concentration of 0.5 µg/mL exhibited a strong anti-dermatophytic potential starting at 24 h after infection of keratinocytes. In fibroblasts, growth of *T. benhamiae* was significantly reduced by a lower concentration after 48 h while a higher TRP concentration was already effective after 24 h (Supplementary Material Fig. S2). However, fungal growth was merely reduced and not fully inhibited.

### Biocompatibility of TRP

The biocompatibility of TRP was initially analysed in HaCaT keratinocytes. 2 µg/mL TRP elicited anti-proliferative effects at 24 h to 72 h, while 5 µg/mL TRP were found to be cytotoxic as indicated by cell viability below 70% accompanied by an absence of proliferating cells. The half maximal lethal concentration (LC_50_)-value determined by the dose-response after 24 h equalled 6.24 µg/mL. Solvent effects could again be excluded (see Supplementary Material Fig. S1). Cytotoxicity (LDH release) was observed at 5 µg/mL after 48 h and 72 h, however, to a much lower extend as expected according to the reduced ATP-derived cell viability (Fig. 1b). The BI referring to IC_50MLN_ equalled 8.83 and 3.99 based on IC_50(ATP-Fungi)_ (Fig. 1c).

Following the initial determination of biocompatibility in HaCaT keratinocytes, analyses with human primary cutaneous cells were carried out with TRP concentrations of 0.5 and 2.0 µg/mL. Primary dermal fibroblasts treated with 2.0 µg/mL TRP showed minor anti-proliferative effects after 48 h and 72 h as indicated by a lower count of viable cell compared to untreated cells, but no LDH release was observed. In primary epidermal keratinocytes an anti-proliferative effect was observed after 48 h already at the lower dosage of 0.5 µg/mL TRP, again no LDH secretion was measured. After 72 h cytotoxic effects were observed resulting in significantly lower viable cell counts and a measurable LDH release (Supplement Material Fig. S3).

### Preventive effect of TRP on *T. benhamiae*-derived cell damage

**Figure 2:**
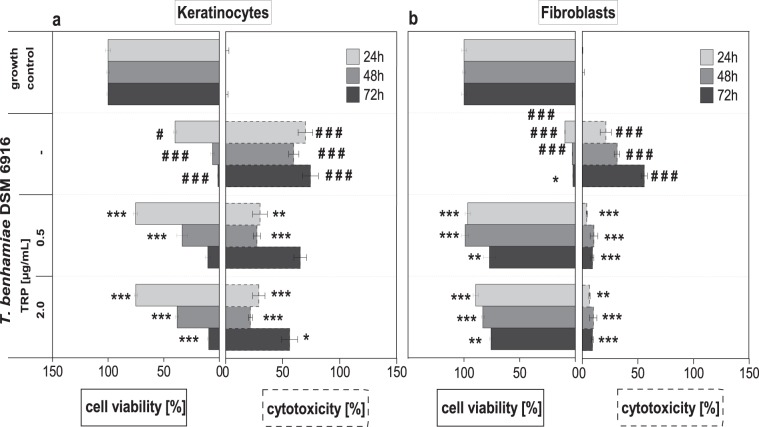
Cell viability and cytotoxicity of epidermal keratinocytes (**a**) and dermal fibroblasts (**b**) after tryptanthrin (TRP) treatment of Trichophyton benhamiae DSM6916-infected cells (n = 3 for keratinocytes, n = 2 for fibroblasts) for 24 h, 48 h and 72 h. Cell viability was analysed by measuring the cellular ATP level and is presented as percentage to growth control (100%). Cytotoxic effects were evaluated by quantifying the LDH release. Cytotoxicity [%] is represented relative to lysis control (100%, data not shown) and growth control (0%). Bars plot the mean ± s.e.m. Statistical analysis was done using the U test figured as *p ≤ 0.05, **p ≤ 0.01 and ***p ≤ 0.001 to the untreated infection control. Hashes depict significant deviations of *T. benhamiae* infection compared to non-infected growth control with p ≤ 0.05 (#), p ≤ 0.01 (##) and p ≤ 0.001 (###).

In epidermal keratinocytes cytotoxic effects of T. benhamiae could not be fully prevented by TRP treatment. Although, LDH secretion was considerably diminished at 24 and 48 h after infection, compared to infected keratinocytes w/o treatment, damage to cells by the fungus was noted at 72 hours (Fig. 2a). Viability of infected keratinocytes treated with 0.5 and 2.0 µg/mL TRP was accordingly significantly increased compared to untreated cells at 24 and 48 hours but diminished considerably after 72 hours (Fig. 2a).

Application of TRP to infected dermal fibroblasts prevented cytotoxic effects even at the low dosage of 0.5 µg/mL. LDH release was significantly reduced compared to infected fibroblasts w/o treatment. In addition, TRP retained cell viability (Fig. 2b).

### Impact of TRP on *T. benhamiae*-induced inflammation

#### Gene expression and protein secretion

**Figure 3:**
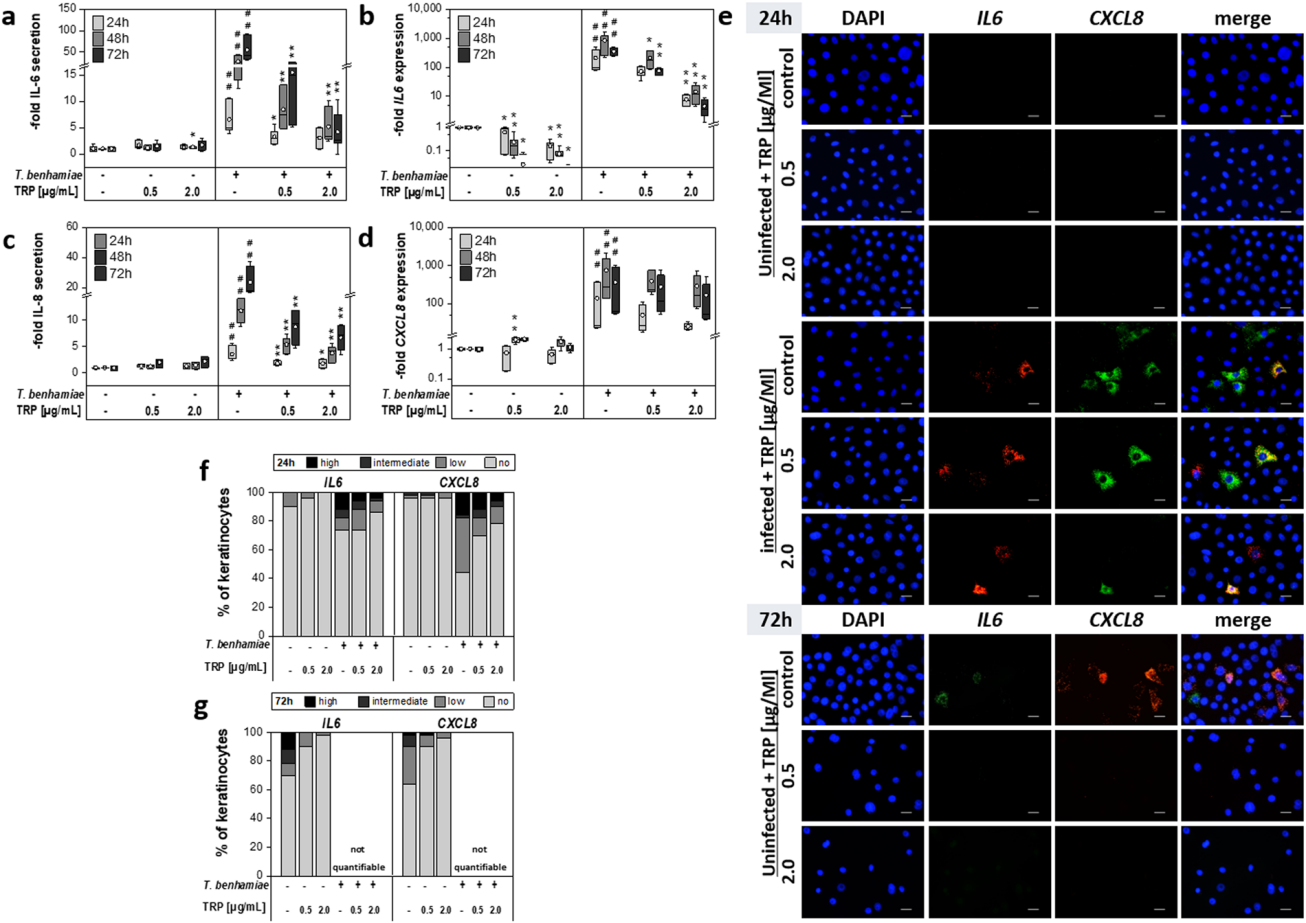
Impact on secretion (**a,c**) and mRNA expression (**b,d**) of pro-inflammatory cytokines interleukin 6 (**a,b**) and interleukin 8 (CXCL8 gene) (**c,d**) after tryptanthrin (TRP) treatment of non-infected and Trichophyton benhamiae DSM6916-infected epidermal keratinocytes (n = 3 experiments). Quantitative analyses of protein secretion (**a,c**) and of gene expression (**b,d**) were performed 24 h, 48 h and 72 h after infection. Relative gene expression was normalized to the housekeeping genes ACTB and TUBB. Data are represented as -fold gene expression or respectively -fold protein secretion compared to unstimulated keratinocytes (growth control) in boxplots comprising median separation, whiskers as minimum and maximum and dots indicating mean values. Statistical analysis was performed using the U test comparing TRP treatment to the corresponding untreated control (*p ≤ 0.05 and **p ≤ 0.01) for uninfected and infected keratinocytes in each partition. Hashes depict significant deviations of *T. benhamiae* infection compared to non-infected growth control with p ≤ 0.05 (#) and p ≤ 0.01 (##). Visualization of IL6 (red) and CXCL8 (green) transcription levels of non-infected and infected epidermal keratinocytes were carried out by mRNA FISH using specific fluorescence-labelled probes (**e**). DAPI was used for nuclear staining (blue). Visual detection of mRNA transcripts failed 72 h post fungal infection due to both insufficient cell permeabilization and mRNA unmasking or an ineffective amplification of the branched DNA because of a very dense fungal growth. Scale bar: 20 µm. For semi-quantification 50 cells per group were categorized according to their number of transcripts. Distribution of the IL6 and CXCL8 mRNA levels after 24 h (**f**) and 72 h (**g**) are summarized in categories of no, low, intermediate or high expression rates. Results represent data of n = 1.

**Figure 4:**
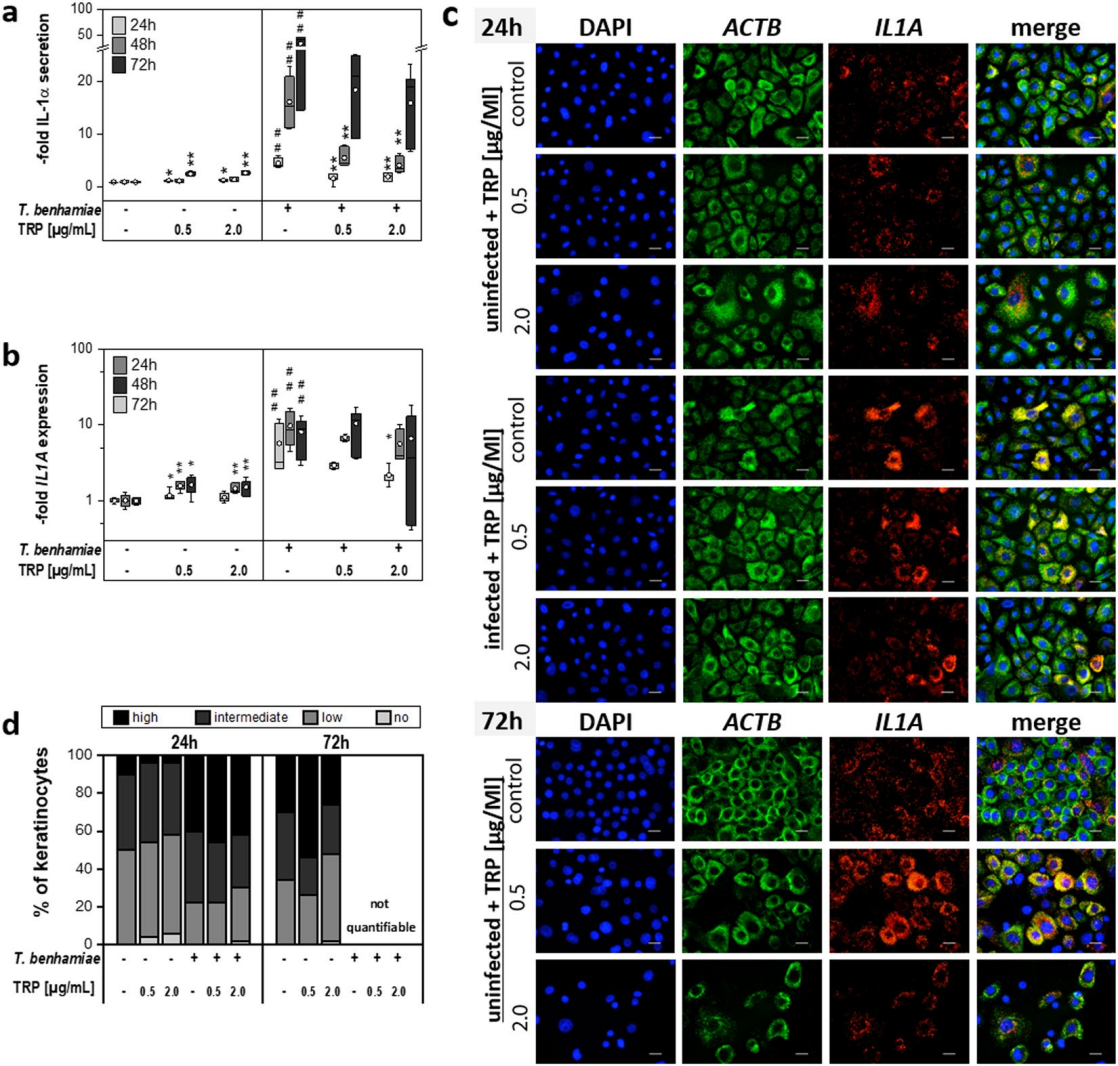
Impact on the pro-inflammatory cytokine interleukin 1 alpha after tryptanthrin (TRP) treatment of non-infected and Trichophyton benhamiae DSM6916-infected epidermal keratinocytes (n = 3 experiments). Quantitative analyses of protein secretion (**a**) and of gene expression (**b**) for the pro-inflammatory cytokine IL-1α were performed 24 h, 48 h and 72 h after infection. Relative gene expression was normalized to the housekeeping genes ACTB and TUBB. Data are represented as -fold gene expression or respectively -fold protein secretion compared to unstimulated keratinocytes (growth control) in boxplots comprising median separation, whiskers as minimum and maximum and dots indicating mean values. Statistical analysis was performed using the U test comparing TRP treatment to the corresponding untreated control (*p ≤ 0.05 and **p ≤ 0.01) for uninfected and infected keratinocytes in each partition. Hashes depict significant deviations of *T. benhamiae* infection compared to non-infected growth control with p ≤ 0.05 (#) and p ≤ 0.01 (##). Visualization of housekeeping gene ACTB and of IL1A transcription levels of non-infected and infected epidermal keratinocytes was carried out by mRNA FISH (**c**). Visualization of housekeeping gene ACTB (green) and of IL1A (red) transcription levels of non-infected and infected epidermal keratinocytes were carried out by mRNA FISH using specific fluorescence-labelled probes. DAPI was used for nuclear staining (blue). Visual detection of mRNA transcripts failed 72 h post fungal infection due to both insufficient cell permeabilization and mRNA unmasking or an ineffective amplification of the branched DNA because of a very dense fungal growth. Scale bar: 20 µm. For semi-quantification 50 cells per group were categorized according to their number of transcripts. Distribution of the IL1A mRNA levels after 24 h and 72 h (**d**) are summarized in categories of no, low, intermediate or high expression rates. Results represent data of n = 1.

Treatment of *T. benhamiae* infected keratinocytes with TRP resulted in a significant down-regulation of genes encoding pro-inflammatory cytokines at high concentrations, in particular of *IL6* (Fig. 3a), but also of *IL1B* and *IL23A* (Table 1). Reduction of *CXCL8* (Fig. 3c) and *CXCL1* was less pronounced in infected keratinocytes. The constitutively expressed cytokines IL-1α (Fig. 4a) and TNF-α (Table 1) were elevated already in early stages of the infection, whereby TRP was able to significantly reduce their gene expression 24 h and 48 h post infection. The cytokine secretion for IL-6, IL-8 (Fig. 3b,d), IL1α (Fig. 4a), TNF-α and especially for IL-1β (Table 1) was also significantly reduced particularly when treated with 2.0 µg/ml TRP over 72 h.

**Table 1:** Expression of genes encoding pro-inflammatory cytokines by epidermal keratinocytes (n = 3 experiments) after tryptanthrin (TRP) treatment of non-infected or Trichophyton benhamiae DSM6916-infected cells.

Epidermal keratinocytes							
-fold gene expression	T [h]	non-infected			*T. benhamiae* DSM6916- infected		
		control	TRP [µg/mL]		control	TRP [µg/mL]	
		—	0.5	2.0	—	0.5	2.0
*IL1A*	24	1.0 ± 0.0	1.2 ± 0.1^**a**^	1.1 ± 0.1	5.7 ± 1.8^**B**^	2.9 ± 0.1	*2.2* ± *0.2*^**a**^
	48	1.0 ± 0.1	**1.6** ± **0.1**^**b**^	**1.4** ± **0.1**^**b**^	9.8 ± 2.0^**B**^	6.8 ± 0.2	5.6 ± 1.2
	72	1.0 ± 0.0	**1.6** ± **0.2**^**a**^	**1.5** ± **0.2**^**b**^	8.1 ± 1.7^**B**^	10.3 ± 2.3	6.6 ± 3.0
*IL1B*	24	1.0 ± 0.1	0.9 ± 0.3	0.9 ± 0.3	2.0 ± 0.4	2.9 ± 1.0	2.2 ± 0.6
	48	1.1 ± 0.1	1.0 ± 0.2	0.6 ± 0.1	2.1 ± 0.2^**B**^	2.9 ± 0.9	2.8 ± 0.6
	72	1.0 ± 0.0	**1.5** ± **0.3**	*0.3* ± *0.0*^**b**^	0.9 ± 0.1	**1.8** ± **0.7**	*0.3 ± 0.1*^***b***^
*TNF*	24	1.0 ± 0.1	**2.1** ± **0.3**^**b**^	**1.6** ± **0.6**	116.5 ± 66.2^**B**^	*50.0 ± 23.1*	*20.7 ± 4.5*
	48	1.0 ± 0.0	**2.4** ± **0.2**^**b**^	**2.0** ± **0.3**^**a**^	188.4 ± 106.0^**B**^	*37.8 ± 15.0*	*87.9 ± 43.5*
	72	1.0 ± 0.1	**2.1** ± **0.6**	1.0 ± 0.3	65.6 ± 32.7^**B**^	46.7 ± 21.1	56.2 ± 31.9
*IL6*	24	1.0 ± 0.0	0.6 ± 0.2^**a**^	*0.2* ± *0.0*^**b**^	209.3 ± 77.4^**B**^	*72.0 ± 12.3*	*7.9 ± 1.4*^***b***^
	48	1.0 ± 0.0	*0.2 ± 0.1*^***b***^	*0.1 ± 0.0*^***b***^	841.0 ± 226.2^**B**^	*210.0 ± 53.2*^***a***^	*14.5 ± 4.1*^***b***^
	72	1.0 ± 0.0	*0.0 ± 0.0*^***a***^	*0.0 ± 0.0*^***a***^	354.8 ± 43.4^**B**^	*75.7 ± 8.1*^***b***^	*4.7 ± 1.3*^***b***^
*CXCL8*	24	1.0 ± 0.0	0.7 ± 0.2	0.7 ± 0.1	141.2 ± 73.5^**B**^	*50.6 ± 17.5*	*25.8 ± 2.2*
	48	1.0 ± 0.0	**2.0** ± **0.2**^**b**^	**1.7** ± **0.2**	755.6 ± 356.8^**B**^	*396.6 ± 115.5*	*294.8 ± 113.6*
	72	1.0 ± 0.1	**2.1** ± **0.1**	1.2 ± 0.1	363.2 ± 191.8^**B**^	277.5 ± 123.6	*169.3* ± *81.0*
*IL23A*	24	1.0 ± 0.0	1.0 ± 0.1	0.6 ± 0.1	14.6 ± 6.6^**B**^	*7.2 ± 2.3*	*2.3 ± 0.4*^***b***^
	48	1.0 ± 0.1	0.8 ± 0.1	*0.4 ± 0.1*^***b***^	40.5 ± 12.1^**B**^	*12.4 ± 1.3*	*10.7 ± 2.5*^***a***^
	72	1.0 ± 0.1	0.9 ± 0.1	*0.4 ± 0.0*^***b***^	21.2 ± 3.8^**B**^	25.2 ± 1.9	11.1 ± 5.0
*CXCL1*	24	1.0 ± 0.0	**1.3** ± **0.2**	0.8 ± 0.1^**a**^	34.0 ± 19.7^**B**^	22.1 ± 11.1	*3.1* ± *0.7*
	48	1.0 ± 0.1	0.8 ± 0.1	*0.5 ± 0.1*^***b***^	29.8 ± 16.5^**B**^	*11.2 ± 4.5*	*9.5 ± 4.1*
	72	1.0 ± 0.1	0.6 ± 0.1^**a**^	*0.2 ± 0.0*^***b***^	25.6 ± 12.4^**B**^	*7.6 ± 3.5*	*7.0 ± 3.9*

Quantitative analyses of gene expression of pro-inflammatory cytokines and chemokines such as interleukin 1 alpha (IL1A), interleukin 1 beta (IL1B), tumour necrosis factor (TNF), interleukin 6 (IL6), interleukin 8 (CXCL8), interleukin 23A (IL23A), and C-X-C motif chemokine ligand 1 (CXCL1) were performed 24 h, 48 h and 72 h after infection. Relative gene expression was normalized to the housekeeping genes ACTB and TUBB. Data are represented as -fold gene expression compared to unstimulated keratinocytes as mean ± s.e.m. Statistical analyses were performed using the U test comparing TRP treatment to untreated non-infected or respectively infected cells (^a^p ≤ 0.05, ^b^p ≤ 0.01 and ^c^p ≤ 0.001) as well as untreated infection control to the growth control (^A^p ≤ 0.05, ^B^p ≤ 0.01 and ^C^p ≤ 0.001). Italic/Underlined represents categorization of a reduced expression rate compared to the respective control (Italic: reduction at least 50%, Underlined: reduction less than 50%, normal: equal). Bold font indicates an elevated expression compared to the respective control.

Application of TRP in non-infected keratinocytes significantly reduced *IL6* (Fig. 3a), *CXCL1* and *IL23A* (Table 1) transcript levels. However, protein secretion of IL-6 was comparable to the untreated growth control (Fig. 3b). IL-1β, TNF-α (Tables 1–2) and IL-8 (Fig. 3c,d) were all unaffected on both protein and transcriptional level compared to the untreated control. Only IL-1*α* was slightly elevated in a TRP-dose-dependent manner (Fig. 4a,b).

**Table 2:** Secretion of pro-inflammatory cytokines by epidermal keratinocytes (n = 3 experiments) after tryptanthrin (TRP) treatment of non-infected or Trichophyton benhamiae DSM6916-infected cells.

Epidermal keratinocytes							
-protein secretion	T [h]	non-infected			*T. benhamiae* DSM6916-infected		
		control	TRP [µg/mL]		control	TRP [µg/mL]	
		—	0.5	2.0	—	0.5	2.0
IL-1α	24	1.0 ± 0.1	1.2 ± 0.0^**a**^	**1.3** ± **0.1**^**a**^	4.6 ± 0.4^**B**^	*1.7* ± *0.4*^**b**^	*2.0* ± *0.3*^**b**^
	48	1.0 ± 0.1	1.2 ± 0.1	**1.5** ± **0.2**	16.2 ± 2.0^**B**^	*5.6* ± *0.8*^**b**^	*4.1* ± *0.7*^**b**^
	72	1.0 ± 0.1	**2.6** ± **0.2**^**b**^	**2.7** ± **0.2**^**b**^	33.7 ± 6.1^**B**^	*18.5* ± *3.0*	*15.9* ± *2.9*
IL-1β	24	1.0 ± 0.5	0.7 ± 0.3	0.9 ± 0.4	31.1 ± 14.2^**B**^	*4.2* ± *1.9*^**a**^	*2.3* ± *0.8*^**a**^
	48	1.0 ± 0.2	*0.4* ± *0.1*	0.6 ± 0.1	108.6 ± 14.1^**B**^	*40.7* ± *6.6*^**b**^	*8.3* ± *1.6*^**b**^
	72	1.0 ± 0.6	1.1 ± 0.6	**2.0 ± 1.2**	264.5 ± 15.0^**B**^	*154.0* ± *7.4*^**b**^	*35.3* ± *5.3*^**b**^
TNF-α	24	1.0 ± 0.3	*0.3* ± *0.1*^**b**^	*0.2* ± *0.0*^**b**^	3.4 ± 0.7^**B**^	*1.2* ± *0.2*^**a**^	*0.7* ± *0.2*^**b**^
	48	1.0 ± 0.2	*0.4* ± *0.1*^**a**^	*0.2* ± *0.0*^**b**^	18.3 ± 4.8^**B**^	9.4 ± 2.5	*5.8* ± *2.3*^**a**^
	72	1.0 ± 0.3	1.0 ± 0.5	2.1 ± 1.1	8.4 ± 4.1^**B**^	6.6 ± 1.7	6.8 ± 3.0
IL-6	24	1.0 ± 0.2	**1.7 ± 0.3**	**1.3 ± 0.2**	6.6 ± 1.3^**B**^	*3.3* ± *0.6*^***a***^	*3.1* ± *0.8*
	48	1.0 ± 0.1	1.2 ± 0.2	**1.3 ± 0.1**^**a**^	27.9 ± 5.5^**B**^	*8.5* ± *1.6*^**a**^	*5.2* ± *1.4*^**b**^
	72	1.0 ± 0.2	**1.5 ± 0.3**	**1.7 ± 0.4**	55.8 ± 11.7^**B**^	*15.5* ± *3.8*^**b**^	*4.2* ± *1.6*^**b**^
IL-8	24	1.0 ± 0.1	**1.3 ± 0.1**	1.3 ± 0.2	3.6 ± 0.6^**B**^	*1.8* ± *0.2*^**b**^	*1.8* ± *0.3*^**a**^
	48	1.0 ± 0.1	1.2 ± 0.1	**1.5 ± 0.3**	11.8 ± 0.9^**B**^	*5.4* ± *0.6*^**b**^	*3.8* ± *0.6*^**b**^
	72	1.0 ± 0.2	**2.0 ± 0.3**	**2.2 ± 0.4**	23.6 ± 3.9^**B**^	*8.7* ± *1.3*^**b**^	*6.7* ± *1.0*^**b**^

Quantitative analyses of cytokine secretion such as interleukin 1 alpha (IL-1α), interleukin 1 beta (IL-1β), tumour necrosis factor α (TNF-α), interleukin 6 (IL-6), interleukin 8 (IL-8) in cell culture supernatants were carried out by ELISA after 24 h, 48 h and 72 h. Cytokine secretion was normalized to 10,000 cells/mL and results are represented as -fold changes of protein secretion compared to unstimulated cells as mean ± s.e.m. Statistical analysis was performed using the U test comparing TRP treatment to untreated non-infected or respectively infected cells (^a^p ≤ 0.05, ^b^p ≤ 0.01 and ^c^p ≤ 0.001) as well as untreated infection control to the growth control (^A^p ≤ 0.05, ^B^p ≤ 0.01 and ^C^p ≤ 0.001). Italic/Underlined represents categorization of a reduced protein secretion compared to the respective control (Italic: reduction at least 50%, Underlined: reduction less than 50%, normal: equal). Bold font indicates an elevated secretion compared to the respective control.

Even at lower concentration, TRP treatment almost entirely reduced the amounts of highly elevated pro-inflammatory cytokines e.g. IL-1α, IL-6 and IL-8 in infected fibroblasts on both transcriptional (Table 3) and protein levels (Table 4). Additionally, the expression of other pro-inflammatory cytokine especially of *IL1B*, *TNF*, *CXCL1* and *IL23A* was also strongly down-regulated in infected fibroblasts (Table 3). TRP treatment of non-infected fibroblasts resulted in a slight elevation of all cytokines on transcript (Table 3) and protein levels (Table 4).

**Table 3:** Expression of genes encoding pro-inflammatory cytokines by dermal fibroblasts (n = 2 experiments) after tryptanthrin (TRP) treatment of non-infected or Trichophyton benhamiae DSM6916-infected cells.

Dermal fibroblasts							
-fold gene expression	T [h]	non-infected			*T. benhamiae* DSM6916-infected		
		control	TRP [µg/mL]		control	TRP [µg/mL]	
		—	0.5	2.0	—	0.5	2.0
*IL1A*	24	1.0 ± 0.0	1.0 ± 0.2	**1.7 ± 0.5**	14.0 ± 4.0^**A**^	*2.1* ± *0.5*^**a**^	*3.1* ± *1.1*^**a**^
	48	1.0 ± 0.0	**3.0 ± 0.2**^**a**^	**2.9 ± 0.3**^**a**^	27.9 ± 8.9^**A**^	*0.9* ± *0.4*^**a**^	*2.7* ± *0.1*^**a**^
	72	1.0 ± 0.1	**3.1 ± 0.3**^**a**^	**2.1 ± 0.2**^**a**^	16.2 ± 1.2^**A**^	*2.0* ± *0.3****c***	*2.7* ± *0.6****c***
*IL1B*	24	1.0 ± 0.1	0.6 ± 0.2	0.6 ± 0.1^a^	4.5 ± 0.4^**A**^	*1.1* ± *0.1*^**a**^	*0.8* ± *0.1*^**a**^
	48	1.0 ± 0.0	1.4 ± 0.4	0.8 ± 0.2	39.2 ± 12.0^**A**^	*2.1 ± 0.2*^**a**^	*1.3 ± 0.3*^**a**^
	72	1.0 ± 0.1	**2.7 ± 0.2**^**a**^	**2.1 ± 0.7**	113.3 ± 11.7^**A**^	*3.8* ± *0.9*^**a**^	*2.2* ± *0.6*^**a**^
*TNF*	24	1.0 ± 0.2	**1.6 ± 0.7**	1.5 ± 1.5	13.0 ± 3.3^**A**^	*5.9* ± *0.6*	11.6 ± 4.3
	48	1.0 ± 0.0	**2.2 ± 1.3**	**3.7 ± 2.9**	15.1 ± 4.1^**A**^	*1.2* ± *0.8*^**a**^	*3.6* ± *0.8*^**a**^
	72	1.0 ± 0.0	1.2 ± 0.5	1.1 ± 0.5	15.4 ± 2.7^**A**^	*0.8* ± *0.5*^**a**^	*0.9* ± *0.3*^**a**^
*IL6*	24	1.0 ± 0.0	0.8 ± 0.1	1.0 ± 0.3	502.1 ± 64.7^**A**^	*28.0* ± *13.9*^**a**^	*29.1* ± *13.2*^**a**^
	48	1.0 ± 0.1	**2.0 ± 0.9**	**1.7 ± 0.3**^**a**^	3728.4 ± 842.1^**A**^	*34.6* ± *20.1*^**a**^	*27.7* ± *11.2*^**a**^
	72	1.0 ± 0.1	**1.4 ± 0.3**	**1.7 ± 0.2**^**a**^	2228.7 ± 564.0^**A**^	*43.4* ± *17.4*^**a**^	*14.5* ± *5.0*^**a**^
*CXCL8*	24	1.0 ± 0.1	**4.1 ± 0.8**^**a**^	**7.7 ± 1.5**^**a**^	73.2 ± 16.2^**A**^	*14.7* ± *4.7*^**a**^	*25.1* ± *5.2*^**a**^
	48	1.0 ± 0.1	**8.7 ± 1.4**	**7.0 ± 1.7**^**a**^	7357.5 ± 791.1^**A**^	*119.2* ± *59.6*^**a**^	*153.8* ± *69.2*^**a**^
	72	1.0 ± 0.1	**5.9 ± 1.5**^a^	**5.1 ± 1.1**^**a**^	12300.1 ± 2464.6^**A**^	*98.3* ± *25.2*^**a**^	*54.1* ± *9.1*^**a**^
*IL23A*	24	1.0 ± 0.1	1.0 ± 0.2	**2.1 ± 0.9**	6.0 ± 2.9	*2.4* ± *0.9*	3.7 ± 1.6
	48	1.0 ± 0.2	0.9 ± 0.2	0.8 ± 0.2	5.3 ± 1.0^**A**^	*1.0* ± *0.2*^**a**^	*1.3* ± *0.3*^**a**^
	72	1.2 ± 0.4	0.8 ± 0.2	1.0 ± 0.2	9.4 ± 0.6^**A**^	*1.0* ± *0.3*^**a**^	*1.1* ± *0.1*^**a**^
*CXCL1*	24	1.0 ± 0.0	**2.3 **±** 0.3**^**a**^	**2.1 **±** 0.7**	2.7 ± 0.5^**A**^	3.7 ± 0.8	**5.1** ± **1.7**
	48	1.0 ± 0.1	**4.5 **±** 0.9**^**a**^	**4.7 **±** 1.0**^**a**^	281.4 ± 32.7^**A**^	*12.2* ± *2.5*^**a**^	*13.3* ± *3.0*^**a**^
	72	1.0 ± 0.1	**2.3 **±** 0.5**^**a**^	**1.5 **±** 0.1**^**a**^	63.0 ± 8.7^**A**^	*4.7* ± *1.0*^**a**^	*5.0* ± *1.4*^**a**^

Quantitative analyses of gene expression of pro-inflammatory cytokines and chemokines such as interleukin 1 alpha (IL1A), interleukin 1 beta (IL1B), tumour necrosis factor (TNF), interleukin 6 (IL6), interleukin 8 (CXCL8), interleukin 23A (IL23A), and C-X-C motif chemokine ligand 1 (CXCL1) were performed 24 h, 48 h and 72 h after infection. Relative gene expression was normalized to the housekeeping genes ACTB and TUBB. Data are represented as -fold gene expression compared to unstimulated fibroblasts as mean ± s.e.m. Statistical analyses were performed using the U test comparing TRP treatment to untreated non-infected or respectively infected cells (^a^p ≤ 0.05, ^b^p ≤ 0.01 and ^c^p ≤ 0.001) as well as untreated infection control to the growth control (^A^p ≤ 0.05, ^B^p ≤ 0.01 and ^C^p ≤ 0.001). Italic/Underlined represents categorization of a reduced expression rate compared to the respective control (Italic: reduction at least 50%, Underlined: reduction less than 50%, normal: equal). Bold font indicates an elevated expression compared to the respective control.

**Table 4:** Secretion of pro-inflammatory cytokines by dermal fibroblasts (n = 2 experiments) after tryptanthrin (TRP) treatment of non-infected or Trichophyton benhamiae DSM6916-infected cells.

Dermal fibroblasts							
-protein secretion	T [h]	non-infected			*T. benhamiae* DSM6916-infected		
		control	TRP [µg/mL]		control	TRP [µg/mL]	
		—	0.5	2.0	—	0.5	2.0
IL-1α	24	1.0 ± 0.1	0.8 ± 0.1	0.6 ± 0.1^**a**^	10.7 ± 2.2^**A**^	*0.7* ± *0.2*^**a**^	*0.8* ± *0.2*^**a**^
	48	1.0 ± 0.2	0.8 ± 0.2	1.0 ± 0.2	108.2 ± 19.2^**A**^	*0.7* ± *0.2*^**a**^	*0.4* ± *0.1*^**a**^
	72	1.0 ± 0.2	0.7 ± 0.2	*0.1* ± *0.1*^**a**^	119.2 ± 16.9	*0.5* ± *0.2*	*0.0* ± *0.0*^**a**^
IL-6	24	1.0 ± 0.1	1.0 ± 0.3	1.0 ± 0.5	38.9 ± 9.0^**A**^	*1.2* ± *0.3*^**a**^	*1.6* ± *0.5*^**a**^
	48	1.0 ± 0.3	1.5 ± 0.7	2.3 ± 1.1	1326.6 ± 237.8^**A**^	*1.9* ± *0.3*^**a**^	*2.3* ± *0.5*^**a**^
	72	1.0 ± 0.2	0.8 ± 0.3	1.1 ± 0.5	1560.9 ± 386.6^**A**^	*2.9* ± *0.5*^**a**^	*2.7* ± *0.6*^**a**^
IL-8	24	1.0 ± 0.3	1.2 ± 0.1	**2.1 ± 0.1**^**a**^	40.7 ± 11.8^**A**^	*1.7* ± *0.0*^**a**^	*3.0* ± *0.3*^**a**^
	48	1.0 ± 0.3	**2.8 ± 0.7**	**4.3 ± 0.6**^**a**^	677.8 ± 156.2^**A**^	*3.4* ± *1.2*^**a**^	*4.7* ± *1.6*^**a**^
	72	1.0 ± 0.4	**1.9 ± 0.3**	**2.6 ± 0.3**^**a**^	636.9 ± 86.8^**A**^	*5.0* ± *1.3*^**a**^	*4.8* ± *0.8*^**a**^

Quantitative analyses of cytokine secretion such as interleukin 1 alpha (IL-1α), interleukin 6 (IL-6), interleukin 8 (IL-8) in cell culture supernatants were carried out by ELISA after 24 h, 48 h and 72 h. Cytokine secretion was normalized to 10,000 cells/mL and results are represented as -fold changes of protein secretion compared to unstimulated cells as mean ± s.e.m. Statistical analysis was performed using the U test comparing TRP treatment to untreated non-infected or respectively infected cells (^a^p ≤ 0.05, ^b^p ≤ 0.01 and ^c^p ≤ 0.001) as well as untreated infection control to the growth control (^A^p ≤ 0.05, ^B^p ≤ 0.01 and ^C^p ≤ 0.001). Italic/Underlined represents categorization of a reduced protein secretion compared to the respective control (Italic: reduction at least 50%, Underlined: reduction less than 50%, normal: equal). Bold font indicates an elevated secretion compared to the respective control.

#### Visualization of transcription levels and distribution

Visual detection of *IL6*, *CXCL8* and *IL1A* mRNA transcripts was performed for epidermal keratinocytes by mRNA FISH analysis. In accordance to the qPCR results, mRNA FISH showed a correspondingly strong down-regulation of *IL6* expression upon TRP treatment in infected keratinocytes in a concentration-dependent manner. In comparison to the qPCR data, visualized *CXCL8* transcript levels were slightly more attenuated in infected keratinocytes after TRP treatment for 24 h (Fig. 3e). After 24 h treatment, TRP had only a slight qualitative impact on the *IL1A* expression of infected cells (Fig. 4c). Under test conditions, a visualization of mRNA level after 72 h was only possible for non-infected keratinocytes. The mRNA FISH assay failed in *T. benhamiae* infected keratinocytes. After repetition it could be concluded that due to the very dense fungal growth, cell permeabilization and mRNA unmasking by protease was insufficient or amplification of the branched DNA was ineffective. Consequently, excess LabelProbe Mix (FITC, Cy3) appeared to interact with fungal mycelium and did not stain the single transcripts (data not shown). TRP application in uninfected cells for 24 h and 72 h resulted in a reduction of *IL6* transcripts after treatment with 0.5 µg/ml TRP and as close as no *IL6* mRNA was found in keratinocytes treated with 2.0 µg/ml TRP after 24 h and 72 h. Similar TRP effects were observed for *CXCL8* (Fig. 3e) and for the constitutively expressed *IL1A* (Fig. 4c).

According to their number of transcripts, cells were quantified categorically to underline the qualitatively observed effects of TRP. The elevated cytokine expression in infected keratinocytes was confirmed by a categorical upward shift of the expressional distribution. A strong inhibitory effect of TRP on *IL6* expression in infected and non-infected keratinocytes was confirmed by the gradual quantification. Rates of *CXCL8* transcripts were also strongly reduced in non-infected cells; however, they were only marginally decreased in infected keratinocytes, consentaneous to qualitative observations (Fig. 3f,g). Further, the slight reduction of *IL1A* by TRP was supported by a proportional upwards shift of cells possessing no and low *IL1A* mRNA in infected and in non-infected cells treated with TRP 2.0 µg/mL after 24 h (Fig. 4d).

### TRP-derived enhancement of AMP expression in keratinocytes

Transcription levels of genes encoding antimicrobial peptides (AMPs) were determined for epidermal keratinocytes as dermal fibroblasts, exhibited no AMP expression previously^21^.

In *T. benhamiae* infected keratinocytes, the elevated gene expression of *RNASE7*, *HBD3* and *HBD2* was further induced by TRP treatment in a dose- and time-dependent manner. Up-regulation of *HBD2* was statistically significant when treated with 2.0 µg/ml TRP after 72 h. Expression level of the psoriasin gene S100A7 was marginally induced after TRP treatment, compared to infected cells w/o treatment where it was significantly down-regulated after 24–48 h. Interestingly, sole application of TRP to keratinocytes also significantly induced gene expression of *HBD2, HBD3* and *RNASE7* (Supplementary Material Fig. S4). Expression levels of psoriasin were found to be much lower than the other AMPs, nonetheless, *S100A7* showed an increase with 0.5 µg/mL TRP by trend.

### The influence of TRP on expression of genes encoding *MKI67* and toll-like receptor 2 (*TLR2*)

Expression of the gene encoding the cell proliferation marker *MKI67* was down-regulated in both cell types after TRP treatment compared to untreated infected cells. DMSO treatment showed little or no effect (Supplementary Material Table S6). Application of TRP led to a reduction of enhanced *TRL2* expression after 24–48 h in a time- and dose-dependent manner (Supplementary Material Fig. S5).

## Discussion

Tryptanthrin (TRP) is one of the active, *Isatis tinctoria-derived* compounds exhibiting strong anti-inflammatory^13–15^ and anti-dermatophytic properties^12,18^. This study, focused on the impact of TRP on the innate immune response of primary cutaneous cells after infection with the zoophilic dermatophyte *Trichophyton benhamiae*. Furthermore, TRP’s preventive and possible immune modulatory effect was investigated.

Preliminary tests using HaCaT keratinocytes and primary cutaneous cells showed that TRP is biocompatible *in vitro*. However, high TRP concentrations led to a decrease in the proliferation of HaCaT keratinocytes as well as primary cells. Further, the mRNA FISH assay pointed out that proliferation of cells treated with a higher dosage of TRP was impaired compared to the untreated growth control over the chosen incubation-time. Hence, it was concluded that TRP possesses anti-proliferative properties. TRP further exhibited antifungal effects in a concentration-dependent manner. Concentrations effective in inhibiting fungal growth were found to be still harmless to cells *in vitro*, as supported by a biocompatibility index (ratio of LC_50_ and IC_50_) greater than one^21^. Therefore, TRP presents a promising candidate for alternative adjuvant therapies to control and combat fungal infections.

In the present study, a previously described dermatophytosis model employing dermal fibroblasts or epidermal keratinocytes infected with *T. benhamiae* DSM6916^20^ was used to investigate the effect of TRP on the fungal-host interaction. As shown before, *T. benhamiae* infection provoked severe cytotoxic effects in both cell types already after 24 h^20^. The tests solely assessed effects of the fungus on cells and not the fungus’ ability to infect fully keratinized tissue. Hnece, the model is limited in terms of using exposed cells in culture media, possibly overestimating harmful effects of the fungus. Hence, future investigations should be performed using full skin models. The application of TRP almost completely prevented *T. benhamiae*–mediated cell damage in dermal fibroblasts, while infection-derived cytotoxic effects on epidermal keratinocytes were only reduced by TRP administration and not fully prevented. This study further investigated TRP’s potential impact on inflammatory events in keratinocytes and fibroblasts. It was found that the enhanced gene expression and secretion of various pro-inflammatory cytokines and chemokines of human keratinocytes and fibroblasts infected with *T. benhamiae* DSM6916^20^ were significantly down-regulated by TRP administration. Thus, it could be assumed that TRP promotes suppression of pathogen-induced acute inflammation. A supporting hypothesis for TRP’s possible mode of action is its ability to target a primary transcription regulator for various cytokines by interacting with suppressors of cytokine signalling (SOCS), which in turn are considered negative regulators of cytokine transcription^22^. Previously, the anti-inflammatory efficacy of the heterocyclic plant metabolites i.e. flavonoids was associated by activation of these SOCS proteins resulting in an inhibition of cytokine production facilitated by suppression of the STAT3 transcription factor^23^. Reduced transcription rates of *IL6*, *CXCL8* and partly of *IL1A* in infected epidermal keratinocytes after 24 h of treatment with TRP were confirmed by visualization and semi-quantification by mRNA FISH analyses in compliance with results determined by RT qPCR analyses. Nevertheless, these results so far do not clarify, whether TRP mediates down-regulation of these cytokines in infected cells by controlling specific signalling pathways or by simply acting as an anti-dermatophytic agent^18^ leading to a lower fungal burden and a consequently weaker inflammatory response. However, the substantial inhibitory effects of TRP were found to be most pronounced towards the IL-6 gene expression and secretion in both cutaneous cell types after *T. benhamiae* infection as well as non-infected cells. Hence, an anti-imflammatory effect of TRP besides the antifungal activity is likely. IL-6 is involved in immune cell regulation, activation, differentiation and mobilization^24^. Additionally, IL-6 plays an essential role in keratinocyte proliferation^25–27^. Hence, the here observed reduction of IL-6 not only reflects the anti-inflammatory action of TRP but also its anti-proliferative properties. This was supported by a down-regulation of the proliferation-marker *MKI67*, observed after administration of TRP in infected and non-infected keratinocytes. These findings are in accordance with the anti-psoriatic action of *Indigo naturalis*, a TRP-containing plant extract used in traditional Chinese medicine^28^. Moreover, in lesional skin of tinea patients, epidermal hyperproliferation is accompanied by a highly significant increase of KI67^29^. In accordance, *MKI67* expression was elevated upon *T. benhamiae* infection in the keratinocyte model used. Administration of TRP reduced transcription of *MKI67*, likely counteracting hyperproliferation caused by *T. benhamiae* infection.

TRP treatment further distinctly reduced IL-1β secretion in *T. benhamiae*-infected keratinocytes, while mRNA levels of IL-1β were nearly unaffected even by a higher TRP dosage over 48 h. Stimulation with the *T. benhamiae* DSM6916 resulted in significant IL-1β production after IL-1β mRNA accumulation, suggesting productive inflammasome activation in epidermal keratinocytes^20^. The observed decrease of cleaved IL-1β protein levels suggests that TRP might interact with inflammasome derived pathways. Han *et al*.^30^ demonstrated that TRP suppressed caspase-1 expression and caspase-1 enzymatic activity in a phorbol myristate acetate/calcium ionophore A23187 (PMACI)-stimulated human mast cell line (HMC-1) *in vitro*. It is known that upon stimulation, the cysteine proteases caspase-1 is activated by the inflammasome^31^ and mediates the cleavage of the inactive precursor of IL-1β and IL-18^32,33^. Hence, inhibition of caspase-1 by TRP could be one possible mode of action explaining the reduced IL-1β protein secretion despite unaffected mRNA levels. Another possible regulation of anti-inflammatory effects of TRP is by inhibiting the degradation of IκBα^34^. The inhibitory molecule IκBα sequesters NF-κB in an inactivate form in the cytoplasm and blocks its translocation to the nucleus^35^, leading to an absent positive feedback for further TNF-α production.

Additionally, TRP treatment could potentially promote the innate immunity of epidermal keratinocytes by differentially up-regulating the expression of antimicrobial peptides (AMPs), especially of *HBD2* and *HBD3*, and marginally of *RNASE7*. The previously reported elevation of such AMPs in *T. benhamiae* infected primary keratinocytes^20^ was even further enhanced for tested β-defensins upon TRP administration. Moreover, the expression of the psoriasin gene *S100A7* was slightly up-regulated in infected keratinocytes after TRP addition, whereas its expression was mostly unaffected in the untreated infection control^20^. AMPs are induced upon danger signals and mediated by pattern recognition receptors (PPRs) such as TLRs or in response to pro-inflammatory cytokines. They not only exhibit potent anti-fungal activity^36–38^ but are actively involved in the innate immune response of the skin, by stimulating chemotactic activity and inducing more cytokines^39^. *T. benhamiae* induced *TRL2* expression 24 h post infection in both cutaneous cell types^20^. Following the recognition of pathogenic patterns, activation of specific TLRs can cause differential up-regulation of the transcription of *HBD2* and *HBD3*^40^ as well as *RNASE7* and *S100A7*^41^. The expression of *HBD2* and *HBD3* is also inducible by TNF-α, IL-1α/-β and IFN-γ^42^, which are further responsible for the induction of constitutively expressed AMPs like *RNASE7*^43^ and *S100A7*^44^. However, the enhanced AMP gene expression in this study especially of *HBD2* and *HBD3* cannot be explained by increased signalling of these cytokines, as they were found to be attenuated by TRP treatment. As *TLR2* was distinctly reduced upon TRP addition, the observed up-regulation of AMPs is not associated with TLR-signalling either. Therefore, AMP induction could be attributed to TRP-derived mechanisms involving transcription processes, which to this point remain unknown and need further investigation.

In conclusion, using a dermatophytosis model employing epidermal keratinocytes or dermal fibroblasts infected with *T. benhamiae* DSM6916, it could be shown that TRP defended cells from severe infection-derived cell damage, by actively counteracting fungal growth. TRP also exhibited potential to reduce pro-inflammatory cytokines on transcriptional and protein levels in both cutaneous cell types under infectious conditions. Interestingly, TRP strongly reduced *IL6* expression in both infected and non-infected keratinocytes, highlighting a potential anti-inflammatory effect and anti-proliferative impact of TRP. The latter was supported by the down-regulation of the proliferation marker *MKI67*, which might be beneficial in tinea-related hyperproliferation. TRP also improves the endogenous host defence by stimulation AMP expression in combat against fungal infection. As all tested AMPs are potentially anti-microbially active, an up-regulation of these AMPs could be one explanation, for the strong anti-microbial properties of TRP during infectious condition by reinforcing the cellular immune response. To the best knowledge, these data show for the first time that the innate anti-microbial response can be triggered by the alkaloid TRP. Hence, this study provides crucial evidence that TRP might be a promising new plant-derived agent applicable for treating inflammatory fungal skin infections due to its strong antifungal properties but also its capability to enhance antimicrobial peptides, reduce pro-inflammatory cytokines and its possible anti-proliferative activities.

## Materials and Methods

### Materials

HaCaT keratinocytes were a gift from Prof. Fusenig, DKFZ (Heidelberg, Germany) and cultivated in DMEM (Dulbecco’s modified Eagle medium, AMIMED® BioConcept Ltd., Allschwil, Switzerland) supplemented with 1% antibiotic-antimycotic solution (10,000 U/mL penicillin, 10,000 µg/mL streptomycin, and 25 mg/mL fungizon, AMIMED® BioConcept Ltd., Allschwil, Switzerland) and 10% foetal bovine serum (PAN-Biotech GmbH, Aidenbach, Germany). Primary epidermal keratinocytes were isolated from human juvenile foreskin and cultivated in keratinocyte growth medium containing 4 µl/ml bovine pituitary extract, 0.125 ng/ml hEGF, 0.33 µg/ml hydrocortisone, 5 µg/ml human insulin, 0.39 µg/ml epinephrine, 10 µg/ml human transferrin, and 0.06 mM CaCl_2_ (PromoCell GmbH, Heidelberg, Germany). This was performed in accordance with relevant guidelines/regulations and approved by the Ethics Committee of the Medical Faculty of the Friedrich-Schiller-University, Jena (4739–03/16). Informed consent was obtained from the legal guardians of all participants. Primary dermal fibroblasts, purchased from Pelo Biotech (Planegg/Martinsried, Germany), were grown in fibroblast growth medium (DMEM, 5 µg/ml recombinant human insulin (AMIMED® BioConcept Ltd., Allschwil, Switzerland), 2% FBS, 5 ng/ml hFGF-2 (Novoprotein Scientific Inc., Summit, USA)). Further, Gibco^®^Trypsin-EDTA (0.05% trypsin-EDTA) was purchased from Thermo Fisher Scientific Inc. (Waltham, USA) and 1x phosphate buffer (w/o Ca^2+^, Mg^2+^) was obtained from AMIMED® BioConcept Ltd. Cultivation of the dermatophyte *Trichophyton benhamiae* DSM6916 (AB088677), procured from the DSMZ – German Collection of Microorganism and Cell Cultures GmbH, was cultured on DERMASEL AGAR (Oxoid^™^, Thermo Fischer Scientific Inc., Waltham, USA). Anti-dermatophytes tests were performed in the liquid culture medium Sabouraud dextrose broth (SDB, Merck Chemicals, Burlington, USA). Tryptanthrin (TRP) was purchased from PhytoLab (Vestenbergsgreuth, Germany). A TRP stock solution of 1 mg/mL was prepared in the organic solvent DMSO (Sigma-Aldrich, Inc. – MERCK, Darmstadt, Germany) and stored at −20 °C.

### Analyses of the bioactivity and biocompatibility of tryptanthrin (TRP)

#### Determination of bio-activity/anti-microbial activity (preliminary tests)

The anti-microbial activity against *T. benhamiae* was analysed according to Finger *et al*.^45^ using the microplate laser nephelometry (MLN) and the ATP bioluminescence assay with appropriate adjustments. Briefly, for MLN a microconidia (MC) suspension was prepared as previously described.^20^ *T. benhamiae* was grown until sufficient sporulation occurred, which is characterized by a mostly grainy to powdery thallus forming after 2–3 weeks particularly on DERMASEL AGAR by Oxoid^™^. MC were extracted from the filamentous fungi by scraping fungal material from the agar with sterile isotonic NaCl solution (Fresenius Kabi AG, Bad Homburg, Germany) and filtering the suspension through an EASYstrainer™ cell sieve (40 µm pore size) to separate MC from larger hyphae fragments. After quantifying the total number of MC using a Neubauer improved counting chamber the inoculum was prepared by adjusting the MC suspension to 10,000 MC/mL. A concentration series of TRP from 0.01 to 10 µg/mL in SDB was added to a 96-well microplate (GreinerBioOne, Frickenhausen, Germany) followed by the inoculum resulting in a final concentration of 100 microconidia/well. Using the NEPHELOstar® Galaxy (BMG Labtech GmbH, Ortenberg, Germany) the microplate was incubated while shaking for 72 h at 30 °C and measurement of the turbidity was done hourly. Subsequently, the half maximal inhibitory concentration (IC_50_) of TRP was determined using the dose-response curve calculation^45^. After 72 h incubation, the fungal ATP content was measured using the microbial viability assay BacTiter-Glo™ (Promega, Mannheim, Germany) and the LUMIstar® Galaxy (BMG Labtech GmbH, Ortenberg, Germany). The percentage of viable fungal cells compared to the untreated growth control was calculated by means of an ATP standard curve and was used to determine of the anti-fungal effect of TRP^45^. Finally, to evaluate whether anti-fungal effects were of fungistatic or fungicide nature, wells with putatively no fungal growth were plated on DERMASEL AGAR for 72 h and observed for re-emerging fungal growth.

#### Biocompatibility towards HaCaT keratinocytes (preliminary tests)

The biocompatibility of TRP was first analysed using HaCaT keratinocytes which were cultivated routinely in 75-cm^2^-cell culture flasks (GreinerBioOne, Frickenhausen, Germany) at 37 °C in a humidified atmosphere containing 5% CO_2_ for 5–7 days. Biocompatibility testing was carried out according to DIN EN ISO 10993-5 as previously described^46^. Briefly, cells were harvested by trypsin-EDTA treatment, seeded into 96-well microplates (GreinerBioOne, Frickenhausen, Germany) with 10,000 cells/well and cultivated in culture medium for 48 h at 37 °C, 5% CO_2_ in a humidified atmosphere. The medium was replaced by either fresh medium (growth control) or a TRP concentration series prepared in culture medium. Triton X-100 (0.1%) served as positive control for cytotoxic effects. Finally, microplates were incubated at 37 °C, 5% CO_2_ in a humidified atmosphere for 1 h, 24 h, 48 h and 72 h. Subsequent analyses included the determination of ATP content (cell viability/ proliferation) and LDH release (cytotoxicity) (see below). The half maximal lethal concentration (LC_50_) of TRP on HaCaT keratinocytes was determined by means of a dose-response curve based on viable cells after 24 h and calculated as previously described^45^. In order to evaluate the biocompatibility index (BI) of TRP the cell compatibility and anti-fungal activity was compared as the ratio of LC_50_ and IC_50_, whereby a BI greater one is favourable indicating high anti-fungal activity with simultaneously good cell compatibility^21^.

#### Biocompatibility towards primary cutaneous cells

Accordingly, to the preliminary tests with HaCaT keratinocytes, the biocompatibility of TRP was further determined for human primary cells such as epidermal keratinocytes and dermal fibroblasts. Analyses of keratinocytes were performed in triplicates and in duplicates for fibroblasts whereas each sample was present in four technical replicates. Determination of cell viability and cytotoxicity was carried out for each of these replicates. Cytokine quantification was carried out using pooled supernatant of four replicates and for gene expression analyses; four replicates of each test condition were pooled to undergo RNA extraction. Subsequently, each pooled sample type was measured in technical duplicates.

#### Measurement of ATP-content

The effect of TRP on the cell viability and proliferation was analysed using the luminometric ATP assay (Perkin Elmer Life Sciences, Waltham, USA) following the manufacturers’ instructions as previously reported^20,47,48^.

#### Measurement of LDH activity

Cell culture supernatants were obtained for determination of lactate dehydrogenase (LDH) activity using a colorimetric assay (Roche, Indianapolis, USA) providing information of *in vitro* cytotoxicity^47^. The assay was run according to the manufacturer’s instructions as previously reported^20^. For evaluation the optical density measured by the microplate photometer FLUOstar® Galaxy (BMG Labtech GmbH, Ortenberg, Germany) was used to determine the *in vitro* cytotoxicity represented in Cytotoxicity [%] relative to positive control (100%) and uninfected growth control (0%).

### Infection of primary cutaneous cells with *Trichophyton benhamiae* and TRP treatment

General cell cultivation and infection were performed as previously described^20^. Primary cells were seeded into 96-well microplates (GreinerBioOne, Frickenhausen, Germany) with 10,000 cells/well and cultivated in keratinocyte growth medium or fibroblast growth medium for 48 h at 37 °C, 5% CO_2_ in a humidified atmosphere. Filtered microconidia suspensions with a total of 10,000 microconidia were added to the cells per well in the respective cell culture medium^20^. For fungal adherence microplates were incubated at 37 °C, 5% CO_2_ in a humidified atmosphere for 1 h before TRP treatment with different concentrations (final concentration 0.5 and 2.0 µg/mL in cell culture medium). As a solvent control, 0.2% DMSO (corresponding concentration for 2.0 µg/mL TRP) was used to clarify TRP specific effects and results are presented in Supplementary Material 1. Cell culture medium was added to the cellular growth control and fungal infection control. Triton X-100 (0.1%) served as control for cytotoxic effects. Finally, microplates were incubated at 37 °C, 5% CO_2_ in a humidified atmosphere for 24 h, 48 h and 72 h. Subsequent analyses included determination of the cell viability (ATP), cytotoxicity (LDH) and inflammation (cytokine secretion) as well as gene expression analyses with n = 3 for keratinocytes and n = 2 for fibroblasts. Treatment effects were determined by comparing infected TRP-treated cells to infected cells w/o TRP treatment as previously published^20^.

#### Measurement cytokine secretion

Co-culture supernatants were stored at −20 °C until cytokine (IL-1α, IL-6, and IL-8) measurements, except for determination of IL-1β and TNF-α, which were processed immediately after collection without freezing because of the rapid degradation of these cytokines. As demonstrated before, IL-1β and TNF-α were not detectable in the supernatant of dermal fibroblasts^20^. Secreted cytokines were measured using commercially available ELISA kits, all according to the manufacturers’ instructions and as previously described^20^. Using the SPECTROstar® Omega plate photometer (BMG Labtech GmbH, Ortenberg, Germany) the absorbance was measured at 450 nm with a reference measurement at 620 nm. Cytokine concentrations were calculated using a 4-parameter-fit standard curve and were normalized to 10,000 cells/mL and expressed as –fold cytokine secretion to the untreated control^20^. For evaluation of treatment effect, results were compared to infected cells w/o TRP treatment.

### Gene expression analysis of primary cutaneous cells

#### RNA extraction, reverse transcription and quantitative real-time reverse transcription PCR (RT qPCR)

Cell lysis, RNA isolation, removal of genomic DNA and reverse transcription were performed according to the previously reported protocol^20^. In brief, the cell lysates were loaded to QIA Shredder spin columns (Qiagen, Venlo, Netherlands), and after centrifugation RNA was purified automatically using the RNeasy^®^ Mini Kit and the QIAcube (Qiagen, Venlo, Netherlands). Genomic DNA was eliminated from the isolated RNA by Ribonuclease assay DNase I, RNase Free (Thermo Scientific™, Waltham, USA). Reverse transcription of a defined RNA concentration was performed by means of the High Capacity cDNA Reverse Transcription Kit by Applied Biosystems (Thermo Scientific™, Waltham, USA). Gene expression analyses were carried out by RT qPCR using the QuantiNova™ SYBR Green PCR Kit (Qiagen, Venlo, Netherlands) and the qTOWER^3^G (Analytik Jena AG, Jena Germany). Data of the relative gene expression was normalized to the housekeeping genes *ACTB* and *TUBB* and are expressed as –fold gene expression compared to the respective untreated cell type. Primer sequences or ordering IDs have been reported previously^20^.

#### mRNA fluorescence *in situ* hybridization

Infected and non-infected epidermal keratinocytes were treated with TRP (0.5 and 2.0 mg/mL) for 24 h and 72 h as described above, however, adjusted to 8-chambered dish format. Subsequently, the mRNA FISH assay (QuantiGene® ViewRNA ISH Cell Assay by Affymetrix, Inc., Thermo Scientific™, Waltham, USA) was performed following the manufacturer’s instructions with previously described modifications^20^. Housekeeping gene *ACTB* served as method control referring to successful unmasking of mRNA targets. Semi-quantification was done by categorizing 50 cells per condition according to their transcription level.

### Quantification of the anti-fungal activity

Calcofluor white (50 µM) staining of the fungal cell wall was used to quantify fungal growth under test conditions. The fluorescence intensity, proportional to the fungal growth, was measured at λ_ex_/λ_em_ 355 nm/460 nm by the Polarstar® Galaxy (BMG Labtech GmbH, Ortenberg, Germany)^20^. The anti-dermatophytic activity of TRP was derived from the fungal growth rate and is presented as the relative anti-dermatophytic activity [%] compared to the untreated infection control (0%).

### Statistics

Regular evaluation and illustration was executed with Excel 2010 (Microsoft, Redmond, USA) and OriginLab 9.0 software (OriginLab Corporation, Northampton, USA). As normal distribution was rejected for the data, the non-parametric Mann-Whitney U test was carried out to assess statistical significances using IBM SPSS 24 statistic software (IBM, Armonk, USA). Asterisks indicate significant deviations (p ≤ 0.05) from the respective untreated growth control or infected control w/o treatment, while differences between growth control and infection control were depicted in hashes (p ≤ 0.05).

## Supplementary information

Supplementary Material.

## Data Availability

All data generated or analysed during this study are included in this published article (and its Supplementary Information files).
