# Supplementary material for: Tryptanthrin promotes keratinocyte and fibroblast responses in vitro after infection with Trichophyton benhamiae DSM6916
Source: Sci Rep. 2020 Feb 5;10:1863. doi: 10.1038/s41598-020-58773-2 (PMC7002663; doi:10.1038/s41598-020-58773-2)
Supplement: Supplementary file 1 — Supplementary Material. [file 41598_2020_58773_MOESM1_ESM.pdf]

Supplementary Material

Tryptanthrin promotes keratinocyte and fibroblast responses *in vitro* after infection with *Trichophyton benhamiae* DSM6916

Authors:
Jana Hesse-Macabata [1], Bianka Morgner[1], Peter Elsner[1], Uta-Christina Hipler[1], Cornelia Wiegand[1]*

Institutions:
[1]Department of Dermatology, Jena University Hospital, Jena, Germany

****Corresponding author:***
Department of Dermatology, University Hospital Jena, Erfurter Straße 35, 07740 Jena, Germany, Email: c.wiegand@med.uni-jena.de, Telephone: +49 3641 9 32 88 78, Fax: +49 3641 9 32 88 75

Effects of tryptanthrins solvent control dimethyl sulfoxide (DMSO)

As solvent control, 0.2 % DMSO (corresponding concentration for 2.0 µg/mL TRP) was used to clarify all TRP specific effects. The following figures and tables demonstrate effects of DMSO compared to the respective untreated controls (growth control and infection control).

Biocompatibility and biofunctionality of DMSO towards HaCaT keratinocytes

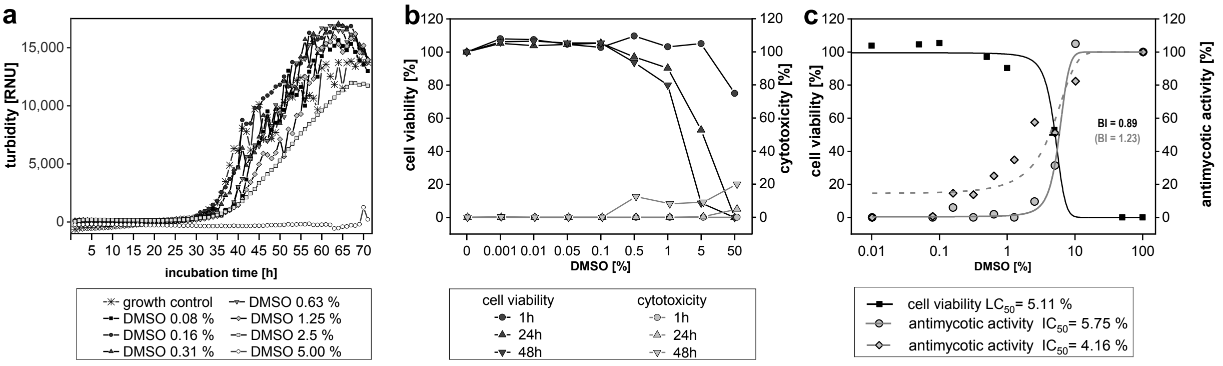

Supplement Figure S1. Antimicrobial activity and biocompatibility of DMSO and evaluation of its biocompatibility index (BI). *The anti-microbial activity against T. benhamiae DSM6916 was analysed by means of the turbidity measurement using the microplate laser nephelometry (MLN). The fungal growth curves are represented in a concentration and time dependent manner (a). The biocompatibility includes the determination of the cell viability and the cytotoxicity using HaCaT keratinocytes treated with concentration series of DMSO for 1h, 24h, and 48h. Cell viability was analysed by measuring the cellular ATP level (black) and is presented as percentage to growth control (100 %). Cytotoxicity [%] was evaluated by quantifying the LDH release (grey) is represented relative to lysis control (100 %, data not shown) and growth control (0 %) (b). The anti-microbial activity was further analysed by quantifying the fungal ATP content using the BacTiter^{TM}-Glo assay (ATP_{fungi}). In order to evaluate the BI of DMSO the dose-response curves of the cell compatibility after 24h (black square) and anti-microbial activity (MLN = grey circle, ATP_{fungi} = grey rhombus) was compared as the ratio of LC_{50} and IC_{50} (c).*

Biocompatibility and biofunctionality of DMSO towards primary cutaneous cells and in the dermatophytosis models

**biocompatibility**		**Dermal fibroblasts**		**Dermal fibroblasts + *T. benhamiae***		
**[%]**	**T [h]**	**-**	**DMSO [%]**	**-**	**DMSO [%]**	
		**-**	**0.2**	**-**	**0.2**	
**viability**	24	100.0 ± 2.2	101.8 ± 3.5	9.3 ± 0.4	6.6 ± 0.8	**a**
	48	100.0 ± 1.2	101.4 ± 2.8	2.8 ± 0.1	0.7 ± 0.3	
	72	100.0 ± 2.4	97.8 ± 1.6	2.2 ± 0.4	0.0 ± 0.2	
**cytotoxicity**	24	0.0 ± 0.4	1.9 ± 0.8	21.1 ± 4.9	19.4 ± 5.1	
	48	0.0 ± 1.9	0.1 ± 0.4	31.2 ± 2.4	28.5 ± 9.4	
	72	0.0 ± 0.2	0.3 ± 0.4	55.7 ± 2.7	60.1 ± 1.9	
**biocompatibility**		**Epidermal keratinocytes**		**Epidermal keratinocytes + *T. benhamiae***		
**[%]**	**T [h]**	**-**	**DMSO [%]**	**-**	**DMSO [%]**	
		**-**	**0.2**	**-**	**0.2**	
**viability**	24	100.0 ± 2.3	**111.9 ± 2.5**	39.7 ± 1.4	**49.6 ± 1.6**	**a**
	48	100.0 ± 1.3	**108.9 ± 1.9**	6.0 ± 1.5	7.9 ± 1.7	
	72	100.0 ± 0.8	**112.6 ± 4.4**	1.3 ± 0.5	1.4 ± 0.4	
**cytotoxicity**	24	0.0 ± 2.9	0.0 ± 0.0	70.0 ± 6.4	**35.4 ± 8.0**	**b**
	48	0.0 ± 0.8	0.0 ± 1.5	59.7 ± 4.4	62.6 ± 6.9	
	72	0.0 ± 2.1	0.0 ± 1.6	74.2 ± 7.0	**56.8 ± 4.6**	**a**

Supplementary Table S1. Cell viability and cytotoxicity (biocompatibility) of dermal fibroblasts (n=2, top) and epidermal keratinocytes (n=3, bottom) after DMSO treatment for 24h, 48h and 72h. *Cell viability was analysed by measuring the cellular ATP level and is presented as percentage to growth control (100 %). Cytotoxic effects were evaluated by quantifying the LDH release. Cytotoxicity [%] is represented relative to lysis control (100 %, data not shown) and growth control (0 %). Data are presented as mean ± s.e.m. Statistical analysis was performed using the U test comparing DMSO treatment to the corresponding untreated control (a: $p \leq 0.05$, b: $p \leq 0.01$ and c: $p \leq 0.001$). Colour highlighting represents categorization of reduced cell viability or respectively an elevated cytotoxicity compared to the respective control (dark grey: at least 50 %, grey: less than 50 %, white: equal). Bold font indicates an elevated cell viability or respectively a reduced cytotoxicity compared to the respective control.*

**biofunctionality**		**Dermal fibroblasts + *T. benhamiae***		**Epidermal keratinocytes + *T. benhamiae***	
	**T [h]**	**-**	**DMSO [%]**	**-**	**DMSO [%]**
		**-**	**0.2**	**-**	**0.2**
**Anti-dermatophytic activity [%]**	24	2.0 ± 1.2	4.7 ± 1.6	5.2 ± 2.4	15.8 ± 2.0
	48	4.0 ± 2.7	**0.4 ± 0.3**	0.6 ± 0.3	0.9 ± 0.5
	72	1.5 ± 0.8	**1.3 ± 1.3**	1.9 ± 0.6	**1.3 ± 0.7**

Supplementary Table S2. Anti-dermatophytic activity (biofunctionality) of DMSO during Trichophyton benhamiae DSM6916 infection of dermal fibroblasts (n=2, left) and epidermal keratinocytes (n=3, right) and for 24h, 48h and 72h. *Analyses were carried out by calcofluor white staining of the chitin of the fungal cell wall and subsequently measuring the fluorescence intensity. The fungal growth rate is expressed as relative fluorescence intensity (RFI) compared to non-infected cells (data not shown). The anti-dermatophytic activity is expressed relative to untreated infected cells (0 %). Data are presented as mean ± s.e.m. Statistical analysis was performed using the U test comparing DMSO treatment to the untreated control (a: $p \leq 0.05$, b: $p \leq 0.01$ and c: $p \leq 0.001$). Colour highlighting represents categorization of an elevated anti-dermatophytic activity compared to the infection control (dark grey: elevation at least 50 %, grey: elevation more than 50 %, white: equal). Bold font indicates a reduced anti-dermatophytic activity compared to the infection control.*

Impact of DMSO on transcription and protein levels in the dermatophytosis models

cytokines		Dermal fibroblasts		Dermal fibroblasts + *T. benhamiae*	
-fold protein secretion	T [h]	-	DMSO [%]	-	DMSO [%]
		-	0.2	-	0.2
IL-1α	24	1.0 ± 0.1	1.0 ± 0.2	10.7 ± 2.2	**20.8 ± 3.7**
	48	1.0 ± 0.2	0.9 ± 0.2	108.2 ± 19.2	**154.2 ± 39.4**
	72	1.0 ± 0.2	0.6 ± 0.1	119.2 ± 16.9	63.3 ± 45.1
IL-6	24	1.0 ± 0.1	0.8 ± 0.3	38.9 ± 9.0	**48.8 ± 11.3**
	48	1.0 ± 0.3	1.1 ± 0.4	1326.6 ± 237.8	1091.3 ± 295.2
	72	1.0 ± 0.2	0.6 ± 0.3	1560.9 ± 386.6	1394.6 ± 485.7
IL-8	24	1.0 ± 0.3	0.6 ± 0.1	40.7 ± 11.8	**53.6 ± 15.2**
	48	1.0 ± 0.3	0.8 ± 0.2	677.8 ± 156.2	**730.8 ± 81.9**
	72	1.0 ± 0.4	0.5 ± 0.1	636.9 ± 86.8	**746.4 ± 73.2**

cytokines		Epidermal keratinocytes		Epidermal keratinocytes + *T. benhamiae*	
-fold protein secretion	T [h]	-	DMSO [%]	-	DMSO [%]
		-	0.2	-	0.2
IL-1α	24	1.0 ± 0.1	1.0 ± 0.1	4.6 ± 0.4	3.5 ± 0.8
	48	1.0 ± 0.1	1.0 ± 0.1	16.2 ± 2.0	14.2 ± 1.8
	72	1.0 ± 0.1	0.9 ± 0.0	33.7 ± 6.1	**46.9 ± 9.4**
IL-1β	24	1.0 ± 0.5	0.9 ± 0.5	31.1 ± 14.2	29.1 ± 11.5
	48	1.0 ± 0.2	0.7 ± 0.1	108.6 ± 14.1	94.4 ± 4.1
	72	1.0 ± 0.6	1.5 ± 0.9	264.5 ± 15.0	272.6 ± 26.3
qTNF-α	24	1.0 ± 0.3	0.7 ± 0.3	3.4 ± 0.7	3.1 ± 1.1
	48	1.0 ± 0.2	0.8 ± 0.2	18.3 ± 4.8	19.1 ± 5.1
	72	1.0 ± 0.3	0.9 ± 0.5	8.4 ± 4.1	**15.4 ± 5.5**
IL-6	24	1.0 ± 0.2	1.3 ± 0.2	6.6 ± 1.3	4.8 ± 1.6
	48	1.0 ± 0.1	1.3 ± 0.2	27.9 ± 5.5	28.8 ± 6.1
	72	1.0 ± 0.2	1.3 ± 0.3	55.8 ± 11.7	28.6 ± 5.0
IL-8	24	1.0 ± 0.1	1.1 ± 0.2	3.6 ± 0.6	2.8 ± 0.6
	48	1.0 ± 0.1	1.1 ± 0.2	11.8 ± 0.9	12.5 ± 1.9
	72	1.0 ± 0.2	1.0 ± 0.2	23.6 ± 3.9	14.7 ± 1.7

Supplementary Table S3. Secretion of pro-inflammatory cytokines by dermal fibroblasts (n=2, top) and epidermal keratinocytes (n=3, bottom) after DMSO treatment of non-infected or Trichophyton benhamiae DSM6916-infected cells. *Quantitative analyses of cytokine secretion such as interleukin 1 alpha (IL-1α), interleukin 1 beta (IL-1β), tumour necrosis factor α (TNF-α), interleukin 6 (IL-6), interleukin 8 (IL-8) in cell culture supernatants were carried out by ELISA after 24h, 48h and 72h. Cytokine secretion was normalized to 10,000 cells/mL and results are represented as -fold changes of protein secretion compared to unstimulated cells as mean ± s.e.m. Statistical analysis was performed using the U test comparing DMSO treatment to the corresponding untreated control (a: $p \leq 0.05$, b: $p \leq 0.01$ and c: $p \leq 0.001$). Colour highlighting represents categorization of a reduced protein secretion compared to the respective control (dark grey: reduction at least 50 %, grey: reduction less than 50 %, white: equal). Bold font indicates an elevated secretion compared to the respective control.*

cytokines		Dermal fibroblasts		Dermal fibroblasts + *T. benhamiae*	
-fold gene expression	T [h]	-	DMSO [%]	-	DMSO [%]
		-	**0.2**	-	**0.2**
IL1A	24	1.0 ± 0.0	0.9 ± 0.3	14.0 ± 4.0	6.7 ± 2.9
	48	1.0 ± 0.0	1.1 ± 0.3	27.9 ± 8.9	13.8 ± 4.9
	72	1.0 ± 0.1	0.5 ± 0.1 a	16.2 ± 1.2	10.8 ± 0.6 b
IL1B	24	1.0 ± 0.1	0.8 ± 0.1 a	4.5 ± 0.4	1.4 ± 0.4 a
	48	1.0 ± 0.0	0.7 ± 0.2	39.2 ± 12.0	11.3 ± 4.2
	72	1.0 ± 0.1	1.1 ± 0.4	113.3 ± 11.7	53.3 ± 16.4 a
TNF	24	1.0 ± 0.2	0.7 ± 0.7	13.0 ± 3.3	13.2 ± 6.0
	48	1.0 ± 0.0	0.0 ± 0.0 a	15.1 ± 4.1	**27.2 ± 11.2**
	72	1.0 ± 0.0	0.5 ± 0.2	15.4 ± 2.7	**25.6 ± 4.0** a
IL6	24	1.0 ± 0.0	0.9 ± 0.2	502.1 ± 64.7	216.5 ± 44.3 a
	48	1.0 ± 0.1	1.3 ± 0.3	3728.4 ± 842.1	3108.1 ± 199.2
	72	1.0 ± 0.1	0.6 ± 0.0 a	2228.7 ± 564.0	1882.3 ± 194.4
CXCL8	24	1.0 ± 0.1	0.6 ± 0.1 a	73.2 ± 16.2	**103.8 ± 43.9**
	48	1.0 ± 0.1	0.8 ± 0.4	7357.5 ± 791.1	5406.6 ± 1255.6
	72	1.0 ± 0.1	1.3 ± 0.3	12300.1 ± 2464.6	**14374.7 ± 889.8**
IL23A	24	1.0 ± 0.1	**1.5 ± 0.4**	6.0 ± 2.9	5.6 ± 2.5
	48	1.0 ± 0.2	0.9 ± 0.3	5.3 ± 1.0	4.2 ± 1.1
	72	1.2 ± 0.4	1.3 ± 0.3	9.4 ± 0.6	**15.5 ± 2.8**
CXCL1	24	1.0 ± 0.0	0.7 ± 0.2	2.7 ± 0.5	1.2 ± 0.1
	48	1.0 ± 0.1	1.2 ± 0.1	281.4 ± 32.7	129.0 ± 10.7
	72	1.0 ± 0.1	0.5 ± 0.1 a	63.0 ± 8.7	34.2 ± 1.9 a

cytokines		Epidermal keratinocytes		Epidermal keratinocytes + *T. benhamiae*	
-fold gene expression	T [h]	-	DMSO [%]	-	DMSO [%]
		-	**0.2**	-	**0.2**
IL1A	24	1.0 ± 0.0	1.0 ± 0.1	5.7 ± 1.8	3.0 ± 0.4
	48	1.0 ± 0.1	1.1 ± 0.0	9.8 ± 2.0	**14.2 ± 3.3**
	72	1.0 ± 0.0	0.9 ± 0.1	8.1 ± 1.7	9.7 ± 2.3
IL1B	24	1.0 ± 0.1	0.6 ± 0.2	2.0 ± 0.4	2.3 ± 0.8
	48	1.1 ± 0.1	0.5 ± 0.1 a	2.1 ± 0.2	2.8 ± 1.0
	72	1.0 ± 0.0	0.5 ± 0.1 b	0.9 ± 0.1	0.7 ± 0.2
TNF	24	1.0 ± 0.1	**1.4 ± 0.3**	116.5 ± 66.2	24.5 ± 6.4
	48	1.0 ± 0.0	**1.6 ± 0.2**	188.4 ± 106.0	**242.2 ± 118.1**
	72	1.0 ± 0.1	1.0 ± 0.2	65.6 ± 32.7	**298.0 ± 170.6**
IL6	24	1.0 ± 0.0	**2.3 ± 0.7**	209.3 ± 77.4	93.7 ± 29.7
	48	1.0 ± 0.0	1.3 ± 0.6	841.0 ± 226.2	**1045.0 ± 253.3**
	72	1.0 ± 0.0	0.3 ± 0.1	354.8 ± 43.4	**585.0 ± 95.2**
CXCL8	24	1.0 ± 0.0	1.3 ± 0.3	141.2 ± 73.5	38.3 ± 10.6
	48	1.0 ± 0.0	**2.0 ± 0.4**	755.6 ± 356.8	**1030.7 ± 454.1**
	72	1.0 ± 0.1	0.8 ± 0.2	363.2 ± 191.8	**1237.4 ± 713.9**
IL23A	24	1.0 ± 0.0	**1.3 ± 0.1** a	14.6 ± 6.6	6.2 ± 1.3
	48	1.0 ± 0.1	**1.4 ± 0.2**	40.5 ± 12.1	46.5 ± 6.6
	72	1.0 ± 0.1	1.0 ± 0.1	21.2 ± 3.8	**43.7 ± 14.3**
CXCL1	24	1.0 ± 0.0	**1.4 ± 0.2**	34.0 ± 19.7	4.3 ± 1.0
	48	1.0 ± 0.1	**1.5 ± 0.2**	29.8 ± 16.5	**80.6 ± 47.1**
	72	1.0 ± 0.1	0.7 ± 0.1	25.6 ± 12.4	**105.2 ± 60.8**

Supplementary Table S4. Expression of genes encoding pro-inflammatory cytokines and chemokines by dermal fibroblasts (n=2, top) and epidermal keratinocytes (n=3, bottom) after DMSO treatment of non-infected or Trichophyton benhamiae DSM6916-infected cells. *Quantitative analyses of gene expression of cytokines such as interleukin 1 alpha (IL1A), interleukin 1 beta (IL1B), tumour necrosis factor (TNF), interleukin 6 (IL6), interleukin 8 (CXCL8), interleukin 23A (IL23A), and C-X-C motif chemokine ligand 1 (CXCL1) were performed 24h, 48h and 72h after infection. Relative gene expression was normalized to the housekeeping genes ACTB and TUBB. Data are represented as -fold gene expression compared to unstimulated cells as mean ± s.e.m. Statistical analyses were performed using the U test comparing DMSO treatment to the corresponding untreated control (a: $p \leq 0.05$, b: $p \leq 0.01$ and c: $p \leq 0.001$). Colour highlighting represents categorization of a reduced expression rate compared to the respective control (dark grey: reduction at least 50 %, grey: reduction less than 50 %, white: equal). Bold font indicates an elevated expression compared to the respective control.*

AMPs		Epidermal keratinocytes		Epidermal keratinocytes + *T. benhamiae*	
-fold gene expression	T [h]	-	DMSO [%]	-	DMSO [%]
		-	0.2	-	0.2
HBD2	24	1.0 ± 0.0	0.3 ± 0.1 a	0.4 ± 0.3	**0.9 ± 0.4**
	48	1.0 ± 0.1	0.3 ± 0.2 a	5.1 ± 2.1	**10.0 ± 3.3**
	72	1.0 ± 0.0	0.5 ± 0.2	10.7 ± 4.9	11.6 ± 4.7
HBD3	24	1.1 ± 0.1	0.9 ± 0.4	0.3 ± 0.1	0.2 ± 0.1
	48	1.2 ± 0.3	**1.5 ± 0.5**	9.2 ± 1.1	9.3 ± 2.7
	72	1.5 ± 0.3	0.8 ± 0.3	8.5 ± 2.9	**33.2 ± 16.9**
RNASE7	24	1.0 ± 0.0	0.9 ± 0.2	8.3 ± 1.4	4.8 ± 0.7
	48	1.0 ± 0.1	0.8 ± 0.1	69.8 ± 23.1	65.0 ± 14.7
	72	1.0 ± 0.0	0.6 ± 0.1 b	33.7 ± 7.3	**66.0 ± 8.6** a
S100A7	24	1.0 ± 0.1	1.0 ± 0.4	0.2 ± 0.1	0.4 ± 0.2
	48	0.9 ± 0.2	0.6 ± 0.2	0.3 ± 0.1	0.1 ± 0.0
	72	1.0 ± 0.0	0.9 ± 0.3	0.1 ± 0.0	0.0 ± 0.0

Supplementary Table S5. Expression of genes encoding antimicrobial peptides (AMPs) by epidermal keratinocytes (n=3) after DMSO treatment of non-infected or Trichophyton benhamiae DSM6916-infected cells. *Quantitative analyses of gene expression of AMPs such as human beta-defensin 2 (HBD2), human beta-defensin 3 (HBD3), ribonuclease 7 (RNASE7) and psoriasin (S100A7) were performed 24h, 48h and 72h after infection. Relative gene expression was normalized to the housekeeping genes ACTB and TUBB. Data are represented as -fold gene expression compared to unstimulated keratinocytes as mean ± s.e.m. Statistical analyses were performed using the U test comparing DMSO treatment to the corresponding untreated control (a: p ≤ 0.05, b: p ≤ 0.01 and c: p ≤ 0.001). Colour highlighting represents categorization of a reduced expression rate compared to the respective control (dark grey: reduction at least 50 %, grey: reduction less than 50 %, white: equal). Bold font indicates an elevated expression compared to the respective control.*

others		Dermal fibroblasts		Dermal fibroblasts + *T. benhamiae*	
-fold gene expression	T [h]	-	DMSO [%]	-	DMSO [%]
		-	0.2	-	0.2
TLR2	24	1.1 ± 0.2	1.1 ± 0.4	2.1 ± 0.7	1.5 ± 0.5
	48	1.0 ± 0.0	1.0 ± 0.1	2.2 ± 0.2	2.3 ± 0.5
	72	1.0 ± 0.1	0.5 ± 0.1 a	1.1 ± 0.2	1.5 ± 0.1
MKI67	24	1.0 ± 0.0	1.3 ± 0.2	0.7 ± 0.1	0.4 ± 0.0 a
	48	1.0 ± 0.1	1.4 ± 0.2 a	2.5 ± 0.4	1.0 ± 0.2 a
	72	1.0 ± 0.1	0.6 ± 0.0 a	1.7 ± 0.3	1.2 ± 0.0
**others**		**Epidermal keratinocytes**		**Epidermal keratinocytes + *T. benhamiae***	
-fold gene expression	T [h]	-	DMSO [%]	-	DMSO [%]
		-	0.2	-	0.2
TLR2	24	1.0 ± 0.1	0.9 ± 0.2	1.6 ± 0.2	**2.3 ± 0.9**
	48	1.0 ± 0.0	0.9 ± 0.2	4.0 ± 2.0	2.0 ± 0.5
	72	1.0 ± 0.1	0.7 ± 0.1	1.1 ± 0.3	1.2 ± 0.1
MKI67	24	1.0 ± 0.0	**1.6 ± 0.1** b	1.9 ± 0.4	1.3 ± 0.3
	48	1.0 ± 0.1	**2.0 ± 0.3** b	0.9 ± 0.2	**2.8 ± 1.6**
	72	1.0 ± 0.0	**1.4 ± 0.5**	0.7 ± 0.2	**3.3 ± 2.0**

Supplementary Table S6. Expression of genes encoding the Toll-like receptor 2 (TLR2) and the proliferation marker MKI67 by dermal fibroblasts (n=2, top) and epidermal keratinocytes (n=3, bottom) after DMSO treatment of non-infected or Trichophyton benhamiae DSM69-infected cells. *Quantitative analyses of gene expression were performed 24h, 48h and 72h after infection. Relative gene expression was normalized to the housekeeping genes ACTB and TUBB. Data are represented as -fold gene expression compared to unstimulated fibroblasts or keratinocytes as mean ± s.e.m. Statistical analyses were performed using the U test comparing DMSO treatment to the corresponding untreated control (a: p ≤ 0.05, b: p ≤ 0.01 and c: p ≤ 0.001). Colour highlighting represents categorization of a reduced expression rate compared to the respective control (dark grey: reduction at least 50 %, grey: reduction less than 50 %, white: equal). Bold font indicates an elevated expression compared to the respective control.*

Supplementary Fig. S2

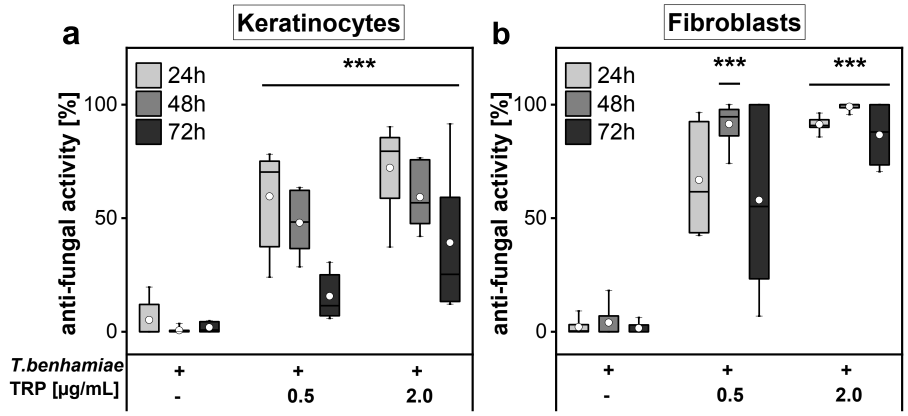

Supplementary Figure S2. Anti-dermatophytic activity of tryptanthrin (TRP) during Trichophyton benhamiae DSM6916 infection of epidermal keratinocytes (a, n=3) and dermal fibroblasts (b, n=2) for 24h, 48h and 72h.

*Analyses were carried out by calcofluor white staining of the chitin of the fungal cell wall and subsequently measuring the fluorescence intensity. The fungal growth rate is expressed as relative fluorescence intensity (RFI) compared to non-infected cells (data not shown). The anti-dermatophytic activity is expressed relative to untreated infected cells (0 %). Results are represented in boxplots comprising median separation, whiskers as minimum and maximum and dots indicating mean values. Statistical analysis was performed using the U test comparing TRP treatment to the untreated infection control (*** $p \leq 0.001$).*

Supplementary Fig. S3

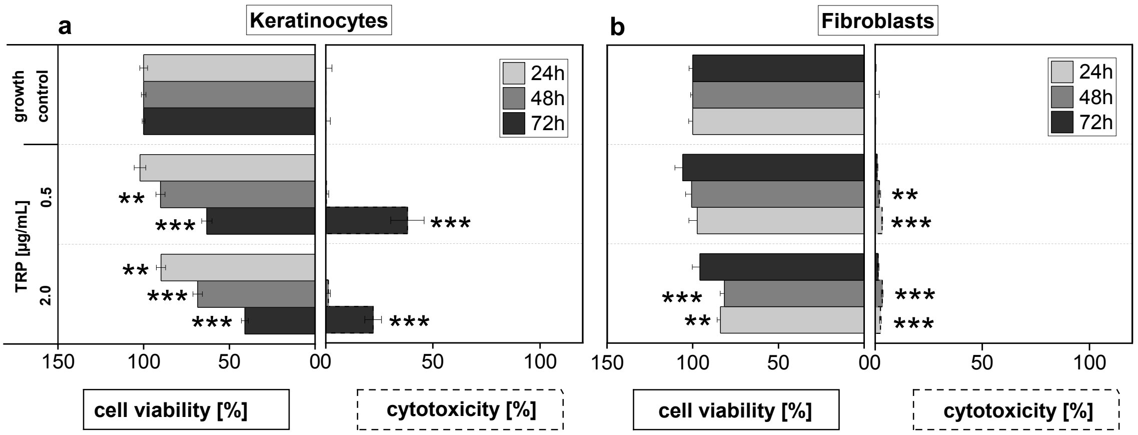

Supplementary Figure S3. Cell viability and cytotoxicity of epidermal keratinocytes (a) and dermal fibroblasts (b) after tryptanthrin (TRP) treatment for 24h, 48h and 72h (n=3 for keratinocytes, n=2 for fibroblasts).

*Cell viability was analysed by measuring the cellular ATP level and is presented as percentage to growth control (100 %). Cytotoxic effects were evaluated by quantifying the LDH release. Cytotoxicity [%] is represented relative to lysis control (100 %, data not shown) and growth control (0 %). Bars plot the mean ± s.e.m. Statistical analysis was done using the U test figured as * $p \leq 0.05$, ** $p \leq 0.01$ and *** $p \leq 0.001$ to the untreated growth control.*

Supplementary Fig. S4

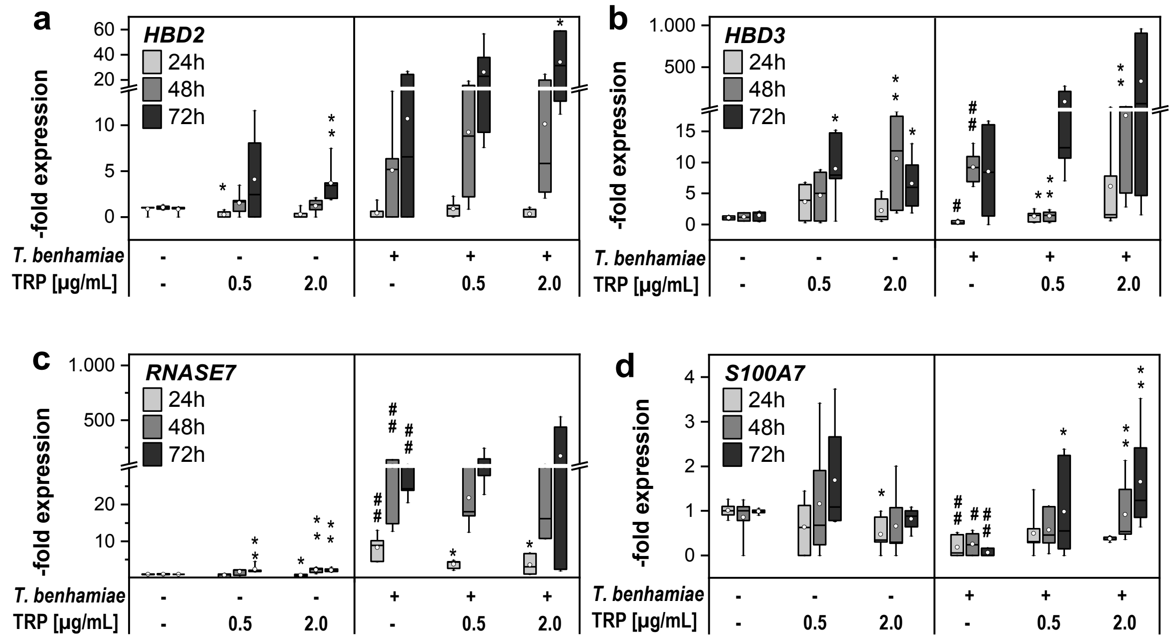

Supplementary Figure S4. Expression of antimicrobial peptides (AMPs) after tryptanthrin (TRP) treatment of non-infected and Trichophyton benhamiae DSM6916-infected epidermal keratinocytes (n=3 experiments).

*Quantitative analyses of gene expression of AMPs such as (a) human beta-defensin 2 (HBD2), (b) human beta-defensin 3 (HBD3), (c) ribonuclease 7 (RNASE7) and (d) psoriasin (S100A7) were performed 24h, 48h and 72h after infection. Relative gene expression was normalized to the housekeeping genes ACTB and TUBB. Data are represented as -fold gene expression compared to unstimulated keratinocytes (growth control) in boxplots comprising median separation, whiskers as minimum and maximum and dots indicating mean values. Statistical analysis was performed using the U test comparing TRP treatment to the corresponding untreated control with * $p \leq 0.05$ and ** $p \leq 0.01$ for uninfected and infected keratinocytes in each partition. Hashes depict significant deviations of T. benhamiae infection compared to non-infected growth control with $p \leq 0.05$ (#) and $p \leq 0.01$ (##).*

Supplementary Fig. S5

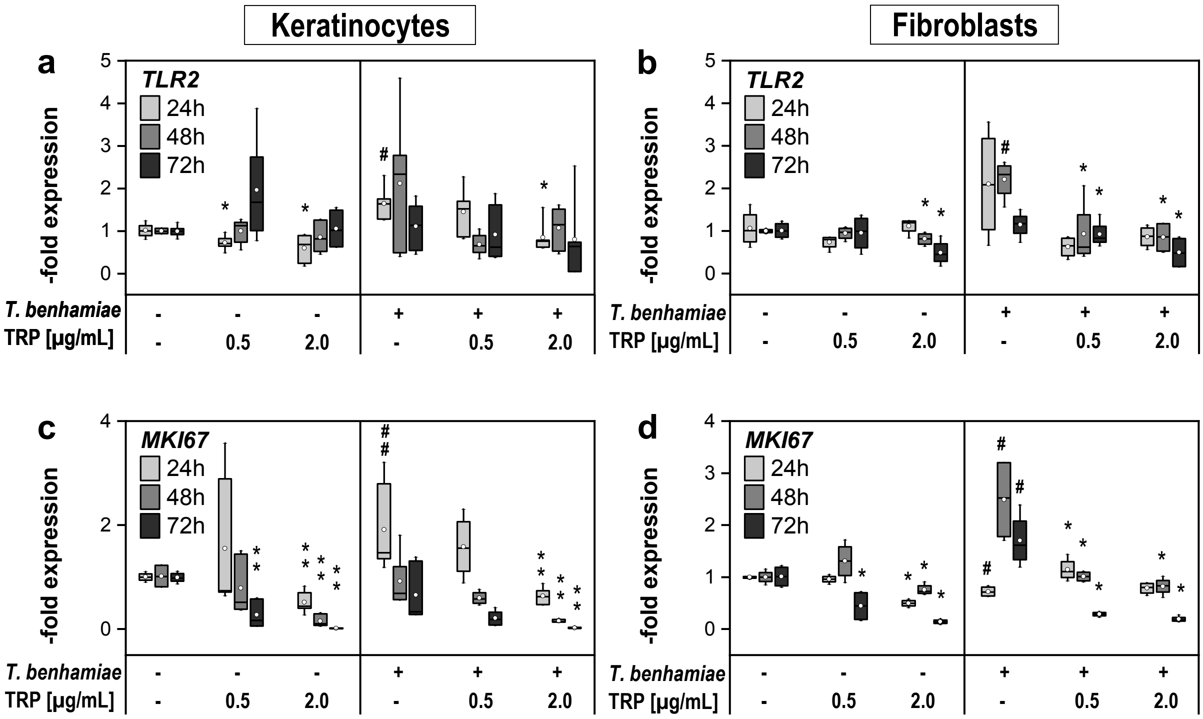

Supplementary figure S5. Expression of genes encoding the pattern-recognition-receptor (a, b) Toll-like receptor 2 (TLR2) and (c, d) the proliferation marker MKI67 after tryptanthrin (TRP) treatment of non-infected and Trichophyton benhamiae DSM6916-infected epidermal keratinocytes (a, c, n=3 experiments) and dermal fibroblasts (b, d n=2 experiments).

*Quantitative analyses of gene expression were performed 24h, 48h and 72h after infection. Relative gene expression was normalized to the housekeeping genes ACTB and TUBB. Data are represented as -fold gene expression compared to unstimulated keratinocytes (growth control) in boxplots comprising median separation, whiskers as minimum and maximum and dots indicating mean values. Statistical analysis was performed using the U test comparing TRP treatment to the corresponding untreated control with * $p \leq 0.05$ and ** $p \leq 0.01$ for uninfected and infected keratinocytes and fibroblasts in each partition. Hashes depict significant deviations of T. benhamiae infection compared to non-infected growth control with $p \leq 0.05$ (#) and $p \leq 0.01$ (##).*
